# A mouse model of autism implicates endosome pH in the regulation of presynaptic calcium entry

**DOI:** 10.1038/s41467-017-02716-5

**Published:** 2018-01-23

**Authors:** Julie C. Ullman, Jing Yang, Michael Sullivan, Jacob Bendor, Jonathan Levy, Ellen Pham, Katlin Silm, Helia Seifikar, Vikaas S. Sohal, Roger A. Nicoll, Robert H. Edwards

**Affiliations:** 1 Departments of Neurology and Physiology, UCSF School of Medicine, San Francisco, CA 94143 USA; 2 Kavli Institute for Fundamental Neuroscience, UCSF School of Medicine, San Francisco, CA 94143 USA; 3 Weill Institute for Neurosciences, UCSF School of Medicine, San Francisco, CA 94143 USA; 4 Graduate Program in Molecular and Cellular Biology, UC Berkeley, Berkeley, CA 94720 USA; 5 Graduate Program in Neuroscience, UCSF School of Medicine, San Francisco, CA 94143 USA; 6 Department of Cellular and Molecular Pharmacology, UCSF School of Medicine, San Francisco, CA 94143 USA; 7 Department of Psychiatry, UCSF School of Medicine, San Francisco, CA 94143 USA

## Abstract

Psychoactive compounds such as chloroquine and amphetamine act by dissipating the pH gradient across intracellular membranes, but the physiological mechanisms that normally regulate organelle pH remain poorly understood. Interestingly, recent human genetic studies have implicated the endosomal Na^+^/H^+^ exchanger NHE9 in both autism spectrum disorders (ASD) and attention deficit hyperactivity disorder (ADHD). Plasma membrane NHEs regulate cytosolic pH, but the role of intracellular isoforms has remained unclear. We now find that inactivation of NHE9 in mice reproduces behavioral features of ASD including impaired social interaction, repetitive behaviors, and altered sensory processing. Physiological characterization reveals hyperacidic endosomes, a cell-autonomous defect in glutamate receptor expression and impaired neurotransmitter release due to a defect in presynaptic Ca^2+^ entry. Acute inhibition of synaptic vesicle acidification rescues release but without affecting the primary defect due to loss of NHE9.

## Abstract (web summary)

The Na^+^/H^+^ exchanger NHE9 is proposed to regulate the H^+^ electrochemical gradient across endosomal membranes. Here, the authors find that NHE9 knockout mice show autism spectrum disorder-like behaviors and disrupted synaptic vesicle exocytosis due to impaired presynaptic calcium entry.

## Introduction

Flux across intracellular membranes generally relies on a H^+^ electrochemical gradient. Neurotransmitters depend on a H^+^ electrochemical gradient for transport into synaptic vesicles. The psychoactive properties of many drugs that act as a weak base to dissipate the chemical component of this gradient (ΔpH) attest to its importance for behavior^1^. The efficacy of amphetamines in the treatment of attention deficit hyperactivity disorder (ADHD) presumably reflects this activity^2^. In addition, antipsychotic compounds accumulate in synaptic vesicles as weak bases, possibly contributing to their therapeutic efficacy^3,4^. The vesicular H^+^ electrochemical gradient thus has an important role in cognition and behavior.

A vacuolar-type H^+^-ATPase creates the H^+^ electrochemical gradient across membranes of the secretory and endolysosomal pathways^5^. Several factors influence the expression of this gradient as either ΔpH or membrane potential (Δψ). The formation of ΔpH generally requires anion entry to relieve inhibition of the H^+^-ATPase by the accumulating positive Δψ, and Cl^−^ is considered the main anion responsible. Intracellular members of the ClC chloride carrier family control acidification in the endolysosomal pathway^6^, but other anions such as the excitatory transmitter glutamate have a similar role in synaptic vesicles^7,8^. It has nonetheless been difficult to understand how differences in anion flux alone could account for progressive acidification from the early endosome to lysosome.

The family of Na^+^/H^+^ exchangers (NHEs) includes plasma membrane isoforms that regulate cytosolic pH, and a subset that localize to intracellular membranes^9,10^. The organellar isoforms exchange cytosolic Na^+^ or K^+^ for lumenal H^+^ and can thus function with anion carriers to determine organelle pH. In addition to effects on ΔpH, the single common yeast ancestor *Nhx1* influences membrane trafficking^11,12^, and a mammalian homolog has been reported to affect endocytosis^13^.

Since many psychoactive drugs also dissipate ΔpH across internal cell membranes, the organellar NHE isoforms might be expected to influence synaptic transmission. Indeed, we previously identified an NHE activity on synaptic vesicles that dissipates ΔpH to promote the Δψ driving glutamate uptake^14^. Studies in culture implicate organellar isoform NHE6 in dendrite morphology^15^, and NHE9 in the trafficking of glial glutamate transporters^16^. However, the physiological role of organellar NHEs in synaptic transmission and behavior has remained unclear.

Recent human genetic studies have implicated the organellar NHEs in neuropsychiatric disease. Recessive mutations in the X-linked endosomal isoform NHE6 produce Christianson syndrome, a developmental disorder with severe intellectual disability and seizures^17^. The condition reflects both endolysosomal dysfunction and a profound defect in neuronal morphology due to reduced signaling by brain-derived neurotrophic factor (BDNF) receptor trkB^15,18^.

The organellar isoform NHE9 has been implicated in ADHD and autism spectrum disorder (ASD). Among the candidate genes identified in a genome-wide association study of ADHD, NHE9 had the highest overall association^19–21^. A condition treated by an agent that dissipates vesicular ΔpH (amphetamine) may thus involve a specific disturbance in the endogenous mechanisms that regulate this gradient. Mutations in NHE9 have also been identified in ASD^22^. ASD form a group of related neurodevelopmental conditions defined by deficits in social interaction (including abnormal communication) and often accompanied by restricted interests, repetitive, stereotyped behavior, and impaired sensory reactivity^23^. Mutations in NHE9 produce seizures as well as ASD^22^. Originally identified in consanguineous families, NHE9 mutations were subsequently found in non-consanguineous families as well, suggesting that heterozygotes can also express the phenotype^24^. Complementation in yeast and astrocytes indicates that the mutations produce a loss of function^16^. In addition, changes in the regulation of NHE6 and NHE9 have been observed more generally in patients with ASD^25^.

We now show that loss of NHE9 in mice reproduces behavior characteristic of ASD, disrupts organelle pH, and impairs synaptic transmission.

## Results

### Generation of NHE9 conditional KO

**Fig. 1:**
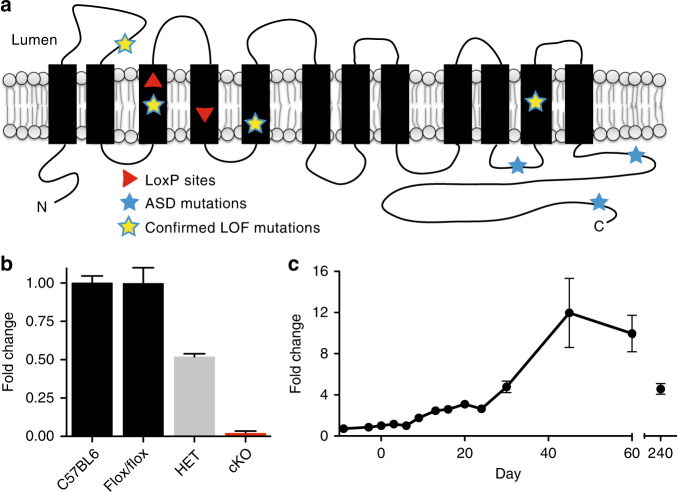
Conditional knockout and developmental expression of NHE9. **a** Diagram shows the 12 predicted transmembrane domains (TMDs) of NHE9 and the location of two loxP sites (red arrowheads) between TMD3 and TMD4. Asterisks indicate the location of ASD-associated mutations. **b** Quantitative RT-PCR demonstrates the reduction in brain NHE9 mRNA from, respectively, heterozygous and conditional knockout (cKO) animals relative to WT and those with unrecombined alleles (flox/flox). Bars indicate the mean and error bars s.e.m. *n* = 2–4 animals/genotype. **c** Time course of brain NHE9 expression (by quantitative RT-PCR) from embryo to adulthood in wild-type C57Bl/6 mice. The data indicate mean ± s.e.m. *n* = 3 mice/time point

To produce a conditional knockout (cKO) of NHE9, we used homologous recombination to introduce loxP sites surrounding exon 5 (Fig. 1a, Supplementary Fig. 1a, b). Deletion of this exon introduces more than a dozen in-frame stop codons through the rest of the transcript. In addition to the full KO, we inactivated NHE9 specifically in the nervous system using the nestin-cre transgene^26^.

To determine whether the cKO eliminates expression of NHE9, we first used the available antibodies. These antibodies recognize the mouse protein over-expressed in heterologous cells, but fail to detect a specific signal in brain extracts from wild-type (WT) mice that is not also present in the NHE9 cKO (Supplementary Fig. 1c). We infer that endogenous NHE9 occurs at levels too low to detect with the available antibodies. However, analysis of NHE9 mRNA by quantitative reverse transcriptase polymerase chain reaction (qRT-PCR) shows expression in the brain with a peak at ~P45 (Fig. 1c). Using RT-PCR, NHE9 mRNA is reduced ~50% in the heterozygote and eliminated in the cKO (Fig. 1b), a finding confirmed with primers spanning the deleted exon (Supplementary Fig. 1d).

### NHE9 cKO mice display behavioral features of ASD

NHE9 cKO animals are born in the expected Mendelian ratios and show normal survival, with several living up to 3 years. The brain weight of cKO mice is the same as WT (Supplementary Fig. 2a). Although a subset of patients with ASD due to mutations in NHE9 have seizures^22^, we only observed generalized tonic–clonic seizures in two animals out of hundreds studied. The phenotype thus appears considerably milder than the ~40% early lethality and prominent epilepsy produced by loss of NHE6^15,18^.

NHE9 cKO mice show no gross defects in motor behavior as assessed by performance on the accelerating rotarod (Supplementary Fig. 2c), by distance traveled, time spent exploring in the open field, or novel object tests (Supplementary Fig. 2e). In addition, they do not differ from heterozygous or WT littermates in the elevated plus maze, open field center quadrant, or novel object exploration, all measures of anxiety (Supplementary Fig. 2e, f). Despite the lack of hyperactivity measured by the open field test, the association of NHE9 with ADHD^19–21^ led us to investigate impulsiveness by the cliff jumping test. However, NHE9 cKO do not differ from WT (Supplementary Fig. 2b), suggesting normal impulse control as well as locomotor activity.

**Fig. 2:**
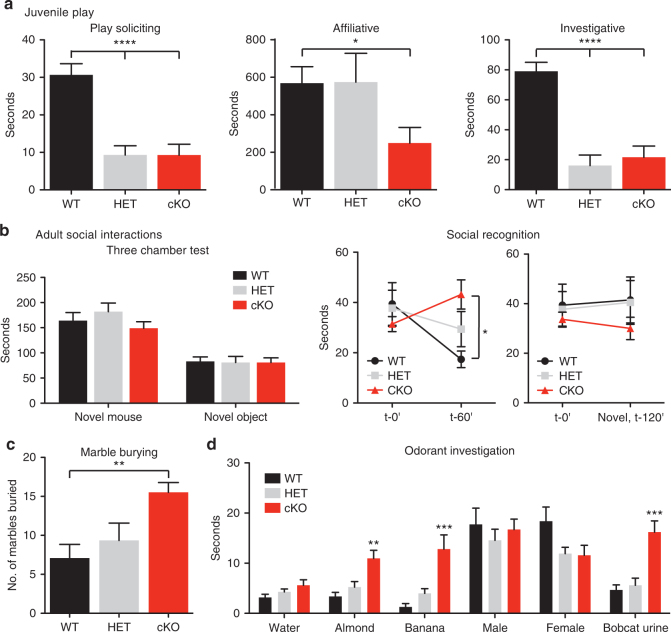
The NHE9 cKO exhibits behavioral features characteristic of ASD. **a** Postnatal day 21 (P21) mice were videotaped for 30 min and the amount of time soliciting play, in affiliative and investigative behavior, were scored. ****p < 0.0001 by one-way ANOVA with Bonferroni multiple comparison. *p = 0.016 by two-tailed Student’s *t* test comparing WT and cKO animals. WT, *n* = 12; HET, *n* = 10; cKO, *n* = 11. **b** Adult NHE9 cKO mice show selective defects in social interaction. Left panel, the three chamber test shows no difference between cKO and WT mice in the intrinsic interest of a novel mouse or novel object; all genotypes spend more time investigating a novel mouse. WT, *n* = 10; HET, *n* = 10; cKO, *n* = 12. Middle panel, NHE9 HET and cKO mice display deficits in social recognition. Total time spent investigating (sniffing, close following) a novel mouse (at t-0′) was compared with the time spent investigating the same mouse 1 h later (t-60′, middle panel) or a novel mouse 2 h later (t-120′, right panel). *p < 0.05 by two-way ANOVA with Bonferroni comparison. WT, *n* = 10; HET, *n* = 10; cKO, *n* = 10. **c** cKO mice exhibit repetitive digging behavior in the marble burying test. The number of marbles buried by each genotype was counted at the end of 30 min. **p < 0.01 by one-way ANOVA with Bonferroni comparison. WT, *n* = 14; HET, *n* = 14; cKO, *n* = 21. **d** NHE9 cKO mice do not distinguish between social, non-social, or aversive odorant cues. **p < 0.01, ***p < 0.001 by one-way ANOVA with Bonferroni comparison. WT, *n* = 10–17; HET, *n* = 10–21; cKO, *n* = 10–28. All bars indicate mean ± s.e.m.

In contrast to this normal behavior, the NHE9 cKO mice exhibit several core features of ASD. First, NHE9 cKO mice exhibit deficits in social interaction. As juveniles, they show considerably reduced play soliciting, such as crawling under and over other mice (Fig. 2a). They also spend less time in affiliative (e.g., allogrooming, huddling) and investigative behavior (e.g., sniffing, following) (Fig. 2a). Consistent with the ASD phenotype observed in non-consanguineous families with rare, presumably heterozygous mutations in NHE9^22^, heterozygous mice also show a defect in play soliciting and investigative behavior. However, the defect in affiliative behavior requires loss of both alleles.

Adult NHE9 cKO mice also show defects in specific social interactions. In the three chamber test, they spend more time in the chamber with a novel mouse than in one with a novel object, and more time with a novel than with a familiar mouse, very similar to WT animals (Fig. 2b). NHE9 cKO mice thus exhibit normal interest in social interactions. In a test for social recognition, WT and heterozygous (HET) mice spend less time investigating a subject mouse upon repeat exposure, but cKO animals fail to habituate, suggesting that they do not recognize the subject (Fig. 2b)^27^. Despite normal interest in social interactions, loss of NHE9 thus specifically impairs social recognition. Interestingly, recent study of a different NHE9 KO mouse focusing on particular behaviors showed a selective defect in preference for social novelty, a test that requires social recognition^28^. The work also showed that loss of NHE9 does not disturb spatial learning in the watermaze. Although social recognition also depends on the hippocampus, it can thus be dissociated from spatial learning^27^.

Second, we investigated NHE9 cKO mice for repetitive and stereotyped behavior characteristic of ASD. cKO animals bury more marbles than WT or HET mice (Fig. 2c), indicating an increase in repetitive digging behavior^29^.

Finally, we examined NHE9 cKO animals for defects in communication and sensory integration. Since rodents rely on olfactory cues to communicate, we tested their innate ability to distinguish social from non-social olfactory cues. In WT mice, exposure to male and female mouse odor elicits a larger response than food odorants (almond, banana) or the aversive odorant bobcat urine (Fig. 2d). In contrast, cKO mice respond equivalently to non-social, social, and aversive scents. However, cKO animals do not differ significantly from WT in the ability to find a buried cookie, arguing against a gross defect in olfaction (Supplementary Fig. 2d). Interestingly, NHE9 is highly expressed in the periglomerular and granule cells of the olfactory bulb^30^. To determine whether loss of NHE9 affects sensory systems more generally, we examined nociceptive responses. Full KO mice show a lower threshold to painful heat, with a trend toward the same effect in cKO animals (Supplementary Fig. 2h). NHE9 may thus be important more broadly for sensory integration. In summary, NHE9 cKO mice reproduce several defining behavioral features of ASD, including deficits in social interaction, repetitive behavior, and impaired sensory integration.

Two recent studies have shown that nestin-cre mice suffer from hypopituitarism and a defect in fear learning, raising the question whether nestin-cre alone accounts for some of the behavioral phenotype observed in the NHE9 cKO^31,32^. Indeed, NHE9 cKO mice weigh less than WT littermates, whereas the full NHE9 KO does not differ from WT (Supplementary Fig. 2h). To test a role for the nestin-cre transgene, we therefore used the full NHE9 KO, without nestin-cre, to re-examine all behavior in which the cKO had shown a phenotype. In almost all cases, we found that the full KO behaved similarly to the cKO (Supplementary Fig. 2h). With the exception of grooming behavior, which showed a difference between WT and cKO but not the full NHE9 KO (Supplementary Fig. 2h), the loss of NHE9 and not the presence of the nestin-cre transgene is thus responsible for the abnormal behavior.

In light of the epilepsy observed in human patients, we monitored the electroencephalogram (EEG) of four KO animals^22^. EEG monitoring reveals recurrent bouts of high-voltage spike activity in all 4 KO mice and 0/4 age-matched WT mice of the same strain (C57Bl/6Tn) as controls (Supplementary Fig. 3a). Spectral analysis indicates that these bouts are characterized by an increase in low-frequency activity (<20 Hz) and concomitant decrease in higher frequency (>30 Hz) activity (Supplementary Fig. 3b–d). They are also associated with immobility. WT animals also show episodes of low-frequency activity, but without the associated high-voltage spikes (Supplementary Fig. 3a, e) or immobility. Although not commonly reported in human patients, high-voltage spike (originally known as spindle) activity lasting <10 s does occur in animal models of absence epilepsy^33,34^. However, the episodes observed in NHE9 KO mice last much longer, up to several minutes. In addition, the peak frequency is 3–4 Hz rather than the 8–12 Hz typically reported (Supplementary Fig. 3e)^33^ and lacks the rhythmic spike-and-wave activity associated with absence seizures. In summary, video EEG demonstrates recurrent epileptiform activity accompanied by immobility, although this does not conform completely to previously described seizure types.

### NHE9 localizes to endosomes and regulates endosomal pH

Since ASD begin early in childhood, we first determined the time course of NHE9 expression, relying on quantitative PCR to detect mRNA transcripts due to the low level of endogenous protein. Figure 1c shows very low levels of brain NHE9 mRNA that increase slowly for 3 weeks after birth, and then rise dramatically 3–6 weeks later, suggesting a role for the protein in the mature brain rather than specifically in development. The Allen Brain Atlas further shows that in the adult, expression of NHE9 appears restricted to the olfactory bulb, superficial cortex, and hippocampus^30^. Since the animals exhibit a defect in social recognition, and social recognition depends on hippocampal function^27^, we have focused on the hippocampus.

**Fig. 3:**
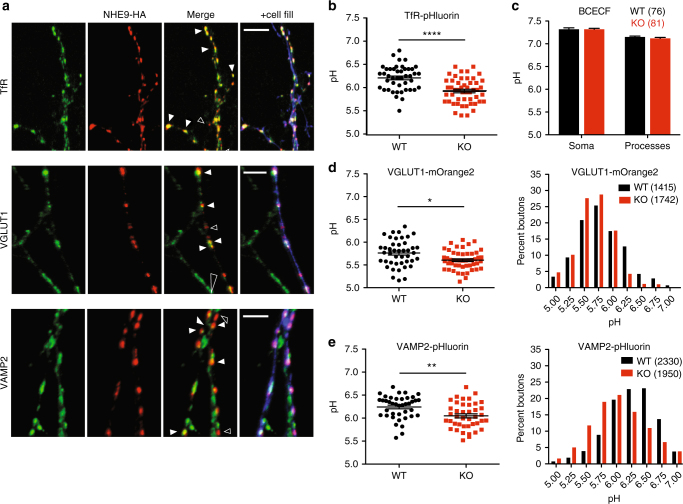
Loss of NHE9 dysregulates the pH of dendritic and axonal endosomes. **a** Dissociated hippocampal cultures prepared from NHE9 KO mice were co-transfected with HA-tagged mouse NHE9 and EGFP (to identify the transfected neurons). NHE9-HA colocalizes strongly with the transferrin receptor (TfR) in dendritic endosomes (Manders coefficient for TfR/HA 0.76 ± 0.07 s.e.m., Figure S3). In axons, NHE9-HA colocalizes with the vesicular glutamate transporter VGLUT1 at some but not all boutons (Manders coefficient for VGLUT1/HA 0.56 ± 0.04 s.e.m. Figure S3). Filled white arrowheads indicate colocalization and unfilled arrowheads HA-NHE9 alone. Scale bars all indicate 5 μm. **b** TfR-pHluorin was transfected into hippocampal neurons and imaged at DIV12–14. Dendritic endosomes expressing TfR-pHluorin exhibit a lower pH in NHE9 KO neurons than WT. ****p < 0.0001 by unpaired two-tailed Student’s *t* test. WT, *n* = 41 neurons/3 cultures; KO, *n* = 41 neurons/3 cultures. **c** Cytosolic pH was imaged in the soma and processes using the pH-sensitive ratiometric dye BCECF (2'-7'-bis(carboxyethyl)-5(6)-carboxyfluorescein). A calibration curve for the dye performed in live neurons (Figure S4) was used to calculate pH. Bars indicate mean ± s.e.m. WT, *n* = 76 neurons/3 cultures; KO, *n* = 81 neurons/3 cultures. **d** VGLUT1-mOrange2 was transfected into hippocampal neurons and imaged at DIV12–14. Boutons containing VGLUT1-mOrange2 exhibit a lower average pH in NHE9 KO neurons than WT. *p < 0.05 by unpaired two-tailed Student’s *t* test. WT, *n* = 43 neurons/3 cultures; KO, *n* = 50 neurons/3 cultures (left panel). Frequency distribution (right panel) of average pH/ bouton for all boutons analyzed in the study, WT, *n* = 1415 boutons/3 cultures; KO, *n* = 1742 boutons/3 cultures. **e** Boutons expressing VAMP2-pHluorin exhibit a lower average lumenal pH in NHE9 KO neurons than WT. **p < 0.01 by unpaired two-tailed Student’s *t* test. WT, *n* = 40 neurons/3 cultures; KO, *n* = 41 neurons/3 cultures (left panel). Frequency distribution (right panel) of average pH/punctae for all punctae analyzed in the study, WT, *n* = 2330 boutons/3 cultures; KO, *n* = 1950 boutons/3 cultures

Previous work in non-neuronal cells has suggested localization of NHE9 as well as NHE6 to endosomes^16,35,36^. Since analysis of the KO showed that none of the available antibodies recognized endogenous NHE9 in the brain (Supplementary Fig. 1c), we transfected an epitope-tagged construct into dissociated hippocampal neurons isolated from neonatal NHE9 KO mice to minimize the potential for mislocalization of the over-expressed protein. To distinguish presynaptic from postsynaptic processes, we used sparse co-transfection with the green fluorescent protein (GFP). Figure 3 and Supplementary Fig. 4b show that in GFP^+^ processes, the epitope-tagged protein colocalizes extensively with transferrin receptor (TfR), which specifically labels dendritic endosomes. The dendritic NHE9-HA does not colocalize with the glutamate receptor subunit GluA2, indicating exclusion of even the over-expressed NHE9 from postsynaptic spines (Supplementary Fig. 4a). The tagged NHE9 also appears in axons, where it partially colocalizes with synaptic vesicle proteins vesicular glutamate transporter 1 (VGLUT1) and vesicular GABA transporter (Fig. 3a, Supplementary Fig. 4b). However, NHE9-HA does not colocalize with all GFP^+^ presynaptic boutons and occasionally labels punctae not expressing the synaptic vesicle proteins. Indeed, it colocalizes better with vesicle-associated membrane protein 2 (VAMP2) than with VGLUT1 (Supplementary Fig. 3b), and VAMP2 does not localize as specifically to synaptic vesicles as VGLUT1^37^. The introduced NHE9 shows only partial colocalization with endogenous NHE6, and less with the lysosomal protein LAMP1 (Supplementary Fig. 4a). In astrocytes, NHE9-HA localizes more clearly to the plasma membrane as well as to intracellular compartments (Supplementary Fig. 4a). The localization suggests that loss of NHE9 may affect the lumenal pH of dendritic endosomes as well as axonal vesicles.

Previous work has suggested a number of roles for the intracellular NHE isoforms. In yeast, *Nhx1* acts as a H^+^ leak to alkalinize endosomes^11^. However, mammals express multiple isoforms with diverse functions. Although heterologous expression has indicated a role for NHE6 and NHE9 in alkalinization^35,38^, NHE7 has recently been suggested to promote vesicle acidification, apparently because it recognizes Na^+^, not K^+^, and the outwardly directed endosome Na^+^ gradient would promote acidification through the mechanism of H^+^ exchange^39^. To determine the role of endogenous NHE9 in regulation of lumenal pH, we took advantage of the ecliptic pHluorin and mOrange2, fluorescent proteins with increased pH sensitivity that can be targeted to specific intracellular compartments by fusion to the lumenal domain of membrane proteins^40,41^. After transfection into cultured neurons, we used buffer at low pH to quench the fluorescence of cell surface protein and ammonium chloride to alkalinize internal membranes, revealing the total pH-sensitive fusion expressed and thereby enabling calculation of lumenal pH^42^. Analysis of pHluorin fused to the lumenal domain of TfR (TfR-pHluorin) shows that the dendritic endosomes of NHE9 KO neurons are more acidic than WT by ~0.3 pH units (Fig. 3b). However, the low pH of synaptic vesicles (~5.8)^42^ would make it difficult to detect hyperacidification using the pHluorin, which has a p*K*a ~7.1^41^. We therefore used a fusion of VGLUT1 to mOrange2 (p*K*a ~6.8)^79^, and observed that loss of NHE9 reduces mean synaptic vesicle pH by ~0.15 units (Fig. 3d). Interestingly, the analysis of pH distribution reveals a selective loss of VGLUT1^+^ punctae with higher lumenal pH (6.25–7.0) (Fig. 3d), suggesting an effect on axonal endosomes rather than typical synaptic vesicles. To address this more directly, we also examined the pH of membranes expressing VAMP2-pHluorin, which distributes more widely in the axon. The analysis shows that loss of NHE9 lowers the pH of VAMP2-pHluorin^+^ membranes by ~0.2 pH units (Fig. 3e). Despite the low expression of NHE9 early in life, loss of the protein thus affects the pH of intracellular membranes in neurons prepared from neonatal animals.

Yeast *Nhx1* has also been suggested to regulate cytosolic pH^11^. We therefore examined cytosolic pH using the ratiometric dye BCECF-AM but found no difference in the pH of cell body or processes of neurons from NHE9 KO and WT mice (Fig. 3c, Supplementary Fig. 4c). NHE9 thus has a specific role at endosomes rather than the plasma membrane.

NHE9 over-expression has also been suggested to influence tumorigenesis through changes in the endolysosomal pathway, but we observed no effect of NHE9 inactivation on ligand-dependent degradation of the δ-opioid receptor expressed in hippocampal neurons (Supplementary Fig. 4d).

### Loss of NHE9 impairs synaptic transmission

**Fig. 4:**
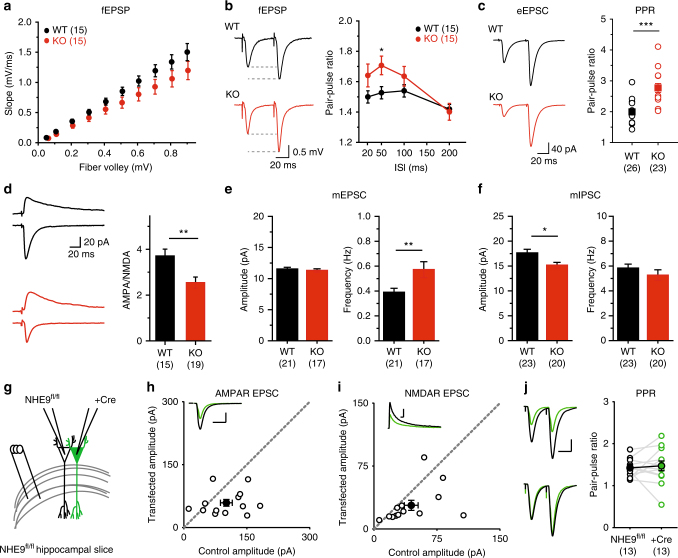
Loss of NHE9 has pleiotropic effects on hippocampal synaptic transmission. **a** fEPSPs recorded in CA1 stratum radiatum are reduced in acute hippocampal slices from NHE9 KO mice. Symbols indicate mean ± s.e.m. *p* = 0.0002 for the difference between genotypes by two-way ANOVA. WT, *n* = 15 neurons/3 mice; KO, *n* = 15 neurons/3 mice. **b** The paired-pulse ratio of fEPSP response at multiple interstimulus intervals (ISIs) shows a significant increase in NHE9 KO slices. Representative traces are shown on the left and mean ± s.e.m. are plotted in the graph at the right. *p < 0.05 by unpaired two-tailed Student’s *t* test. WT, *n* = 15 neurons/3 mice; KO, *n* = 15 neurons/3 mice. **c** Whole cell recording of evoked EPSCs from pyramidal neurons in CA1 also shows increased PPR at 50 ms ISI in NHE9 KO slices. ****p < 0.0001 by unpaired two-tailed Student’s *t* test. WT, *n* = 26 neurons/4 mice; KO, *n* = 23 neurons/4 mice. **d** AMPA/NMDA ratios from WT (*n* = 15 neurons/3 animals) and KO (*n* = 19 neurons/4 animals) are compared. **p < 0.01. **e** Whole cell recordings from CA1 neurons of NHE9 KO mice show normal mEPSC amplitude but increased frequency. **p = 0.0098, F-test *p* = 0.01 by unpaired two-tailed Student’s *t* test. WT, *n* = 21 neurons/4 mice; KO, *n* = 17 neurons/4 mice. **f** mIPSC amplitude is reduced and the frequency unchanged in NHE9 KO neurons. *p* = 0.011 by unpaired two-tailed Student’s *t* test. WT, *n* = 23 neurons/4 mice; KO, *n* = 20 neurons/4 mice. **g** Schematic of single cell KO recording of NHE9 in cKO slices. **h**, **i** NHE9 deletion caused equivalent reductions in AMPAR and NMDAR transmission as compared to the simultaneously recorded control neuron. WT, *n* = 13 neurons/3 mice; KO, *n* = 13 neurons/3 mice; *p < 0.05 by paired Student’s *t* test. **j** No change in paired-pulse ratio was detected in the postsynaptic KO neuron, WT, *n* = 13 neurons/3 mice; KO, *n* = 13 neurons/3 mice

The dysregulation of dendritic and axonal endosome pH raised the possibility of direct effects on synaptic transmission. We therefore examined the synapses formed by Schaffer collaterals in stratum radiatum of hippocampal region CA1, where both presynaptic CA3 and postsynaptic CA1 pyramidal cells express NHE9^30^. In acute hippocampal slices, the NHE9 KO shows a small but significant reduction relative to WT in field excitatory postsynaptic potentials (fEPSPs) normalized to the axonal fiber volley (Fig. 4a). The paired-pulse ratio also increases, consistent with a reduced probability of vesicle release (Fig. 4b). Whole cell recording of evoked excitatory postsynaptic currents (EPSCs) confirms the increased paired-pulse ratio (Fig. 4c).

In addition, the NHE9 KO shows a reduction in the ratio of α-amino-3-hydroxy-5-methyl-4-isoxazolepropionic acid (AMPA) receptor to *N*-methyl-d-aspartate (NMDA) receptor activation (Fig. 4d). Although this ratio is generally considered to reflect a change in postsynaptic responsiveness to glutamate, it might also reflect a change in the amount of glutamate released per vesicle. Since synaptic vesicles exhibit an NHE-like activity that affects vesicular glutamate transport^14^, we also examined the effect of the NHE9 KO on vesicle filling. However, the KO does not affect glutamate uptake by purified synaptic vesicles (Supplementary Fig. 5d). Thus, NHE9 does not contribute to the synaptic vesicle NHE activity previously reported^14^.

We also examined spontaneous transmitter release. In the absence of NHE9, miniature EPSCs (mEPSCs) show increased frequency but normal amplitude (Fig. 4e). Since the response to paired-pulse stimulation indicates reduced release probability, the increase in mEPSC frequency might reflect an increase in synapse number. However, the morphology of hippocampal neurons in NHE9 KO animals appears normal by Nissl stain (Supplementary Fig. 5a), and the Golgi stain shows normal spine density in CA1 stratum radiatum (Supplementary Fig. 5b, c). Thus, mEPSC frequency increases despite reduced evoked release and no apparent change in synapse number, indicating divergent effects of the NHE9 KO on spontaneous and evoked release. Miniature inhibitory postsynaptic currents (mIPSCs) show a reduction in amplitude without a change in frequency (Fig. 4f), suggesting a change in GABA receptor expression or vesicular GABA transport, although we did not observe a difference in GABA uptake by synaptic vesicles from the NHE9 KO (Supplementary Fig. 4d).

To determine whether the effects on synaptic transmission result from changes in circuit activity, we took advantage of the conditional KO to inactivate NHE9 in individual CA1 neurons. Organotypic hippocampal cultures from NHE9^fl/fl^ mice were biolistically transfected at low density with Cre recombinase and GFP. Eighteen days later, we stimulated Schaffer collaterals and performed simultaneous whole cell recording from transfected and neighboring-untransfected control neurons (Fig. 4g). NHE9 deletion causes an equivalent reduction in AMPA and NMDA responses relative to the adjacent control (Fig. 4h, i). In contrast to the full KO (Fig. 4b, c), the loss of postsynaptic NHE9 did not affect release probability as determined by the paired-pulse ratio (Fig. 4j). Thus, loss of NHE9 causes a cell-autonomous effect on glutamate receptors, and the effect on release probability does not simply reflect retrograde signaling from a postsynaptic neuron lacking NHE9, suggesting a cell-autonomous presynaptic role as well.

### Loss of NHE9 impairs synaptic vesicle exocytosis

**Fig. 5:**
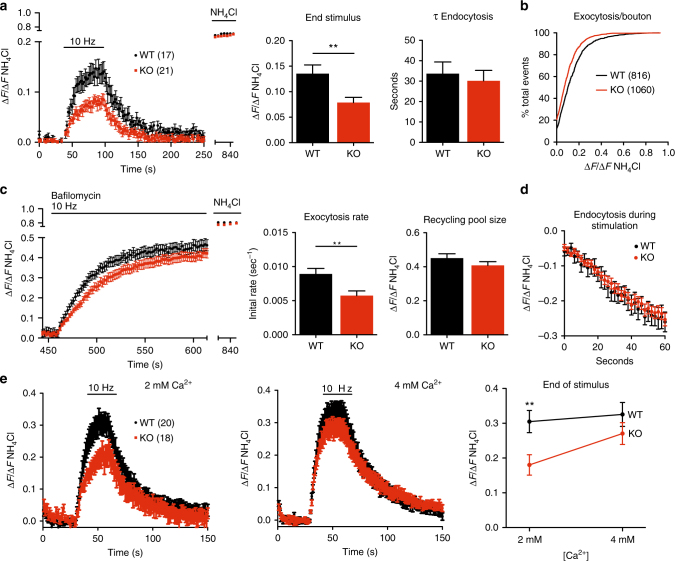
Loss of NHE9 impairs synaptic vesicle exocytosis. **a** Cultured hippocampal neurons from NHE9 KO mice show a smaller VGLUT1-pHluorin fluorescence response than WT cells to 10 Hz stimulation for 60 s (left panel). Middle panel indicates fluorescence at the end of stimulation. **p < 0.01 by unpaired Student’s *t* test. Right panel shows no change in the time constant of endocytosis (*τ*), *p* = 0.65. **b** Cumulative frequency distribution of the extent of exocytosis for all boutons shows a consistent shift to smaller values for the NHE9 KO relative to WT. *p* < 0.0001 by Kolmogorov–Smirnov. WT, *n* = 816 boutons; KO, *n* = 1060. **c** Hippocampal neurons expressing VGLUT1-pHluorin were stimulated at 10 Hz for 60 s, then stimulated again at 10 Hz for 150 s in the presence of bafilomycin. The fluorescence change in the presence of bafilomycin (left) was used to determine the initial rate of exocytosis (middle), which is reduced for NHE9 KO neurons relative to WT, *p* = 0.0047. The recycling pool size, determined by normalizing the peak fluorescence response in bafilomycin to the total fluorescence revealed in NH_4_Cl (right), shows no change (*p* = 0.22). **d** The rate constant of endocytosis during stimulation was calculated by subtracting the fluorescence in the presence of bafilomycin from that in the absence. NHE9 KO neurons do not differ from WT in endocytosis during or after the stimulus. *n* = 17 (WT) or 21 (KO) coverslips/genotype for **c** and **d**. Bar graphs indicate mean ± s.e.m. **e** Response of VGLUT1-pHluorin to 10 Hz stimulation shows that 4 mM Ca^2+^ rescues the exocytosis defect in the NHE9 KO neurons observed at 2 mM Ca^2+^, **p < 0.01 by two-tailed unpaired *t* test. *N* = 20 WT, 18 KO coverslips

To characterize the defect in neurotransmitter release suggested by the increased paired-pulse ratio, we again took advantage of the ecliptic pHluorin fused to the lumenal domain of synaptic vesicle protein VGLUT1. Quenched at the low pH of synaptic vesicles, the protein increases in fluorescence when exposed to the higher external pH by exocytosis, then reacidifies rapidly after endocytosis^43^. Transfected into dissociated hippocampal neurons, the reporter shows substantially reduced peak fluorescence response to stimulation at 10 Hz in cells from the NHE9 KO than from WT mice (Fig. 5a), suggesting a defect in synaptic vesicle exocytosis. The impairment does not involve an identifiable subset of boutons, but rather affects the entire range including those with both large and small responses (Fig. 5b). It is important to note that the increased release of H^+^ from hyperacidic synaptic vesicles is unlikely to account for the reduced pHluorin response in dissociated cultures because the pHluorin fusion remains on the cell surface much longer than required to clear H^+^, which is not limited by diffusion, and the fluorescence in KO (or WT) neurons does not increase when stimulation stops. Although NHE activity might influence the rate of synaptic vesicle reacidification measured with the pHluorin after stimulation, the rate of fluorescence decay also appears unchanged by loss of NHE9 (Fig. 5a), excluding effects on endocytosis as well as reacidification.

Since the response of VGLUT1-pHuorin to stimulation reflects a combination of both exocytosis and endocytosis, we also used the H^+^-ATPase inhibitor bafilomycin to block the reacidification of recycling synaptic vesicles, thereby preventing the decrease in fluorescence that accompanies endocytosis, and allowing us to focus specifically on synaptic vesicle exocytosis. Using bafilomycin, we found that neurons from the NHE9 KO respond more slowly than WT to stimulation (Fig. 5c). By subtracting the fluorescence response in the absence of bafilomycin from that in the presence of the drug, we also measured the endocytosis that occurs during stimulation^44^. Boutons of the NHE9 KO show no difference from WT in endocytosis during the stimulus (Fig. 5d). NHE9 thus specifically affects synaptic vesicle exocytosis.

### NHE9 influences presynaptic calcium entry

How does the loss of NHE9 impair synaptic vesicle exocytosis? It might affect the pool of synaptic vesicles available for release, also known as the recycling pool^45^. However, the recycling pool detected by stimulation of cells expressing VGLUT1-pHluorin in the presence of bafilomycin and normalized to the total vesicle pool revealed in NH_4_Cl shows no difference between NHE9 KO and WT (Fig. 5c). Release also reflects the number of synaptic vesicles docked and primed for release, known as the readily releasable pool (RRP). Using stimulation at 20 Hz for 2 s to monitor the size of the RRP, we find no effect of the NHE9 KO on RRP size (Supplementary Fig. 6a). We also examined a wide range of presynaptic as well as postsynaptic proteins by quantitative western analysis of hippocampal extracts (Supplementary Fig. 6b). For all of these proteins, the NHE9 KO shows no difference from WT.

In the absence of a change in pool size or synaptic protein expression, we considered that a change in calcium entry or sensitivity might account for the role of NHE9 in synaptic vesicle exocytosis. We therefore examined the VGLUT1-pHluorin response to stimulation in normal (2 mM) and high (4 mM) Ca^2+^. Figure 5e shows that 4 mM Ca^2+^ rescues the exocytosis defect in NHE9 KO neurons.

**Fig. 6:**
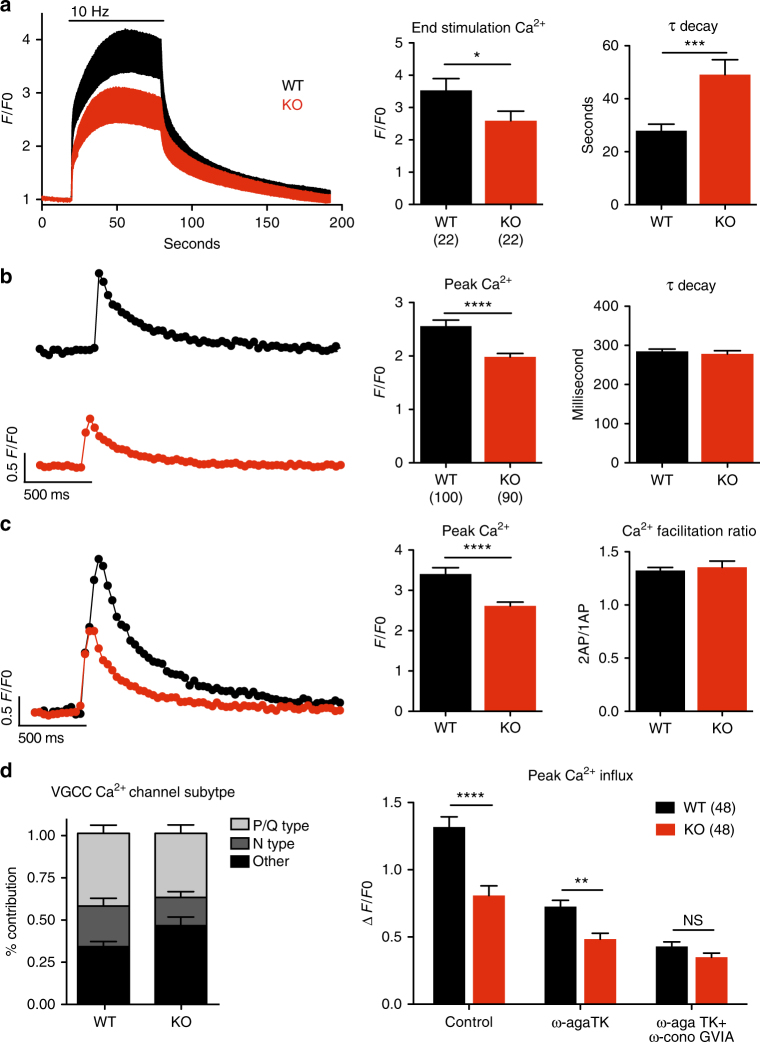
Reduced calcium influx in the boutons of NHE9 KO mice. **a**, **b** Cultured hippocampal neurons were loaded with Fluo5F-AM and stimulated at either 10 Hz for 60 s (**a**) or once (**b**). Left panels show the mean response for all coverslips and the middle panel the mean fluorescence during the final 10 s of stimulation. The decay for each curve was fit to a single exponential and the mean time constant for each coverslip plotted in the right panel. Bars indicate mean ± s.e.m. *p < 0.05, ***p < 0.001, and ****p < 0.0001 by unpaired two-tailed Student’s *t* test. *n* = 22 coverslips/3 cultures/genotype for **a** and *n* = 90–100 action potentials averaged from 30 boutons/coverslip across 14–15 coverslips and 3 cultures/genotype for **b**. **c** The same boutons shown in **b** were stimulated twice at a 50 ms interval. Left panel shows sample traces, middle panel the mean peak fluorescence, and right panel the ratio of paired to single action potential responses (**b**). Bars indicate mean ± s.e.m. ****p < 0.0001 by unpaired two-tailed Student’s *t* test. *n* = 90–100 responses averaged from 30 boutons/coverslip across 14–15 coverslips and 3 cultures/genotype. **d** Hippocampal neurons were stimulated to produce single action potentials and bouton Ca^2+^ entry monitored with Fluo5F in the presence of first ω-Agatoxin TK (300 nM) to block P-/Q-type Ca^2+^ channels and then omega Conotoxin GVIA (1 μM) to block N-type channels as well. NHE9 KO neurons show less presynaptic Ca^2+^ entry through P-/Q- and N-type channels than WT neurons (right panel). Left panel depicts the contribution of each Ca^2+^ channel subtype to the total response. Bars indicate mean ± s.e.m. ****p < 0.0001, **p<0.01 by paired two-way ANOVA. *n* = 48 paired action potentials averaged from 30 boutons/coverslip across 12 coverslips and 3 cultures/genotype

Rescue of the defect in transmitter release by increased external Ca^2+^ suggests that loss of NHE9 impairs either Ca^2+^ entry or sensitivity. Since the amount of SNARE proteins and Ca^2+^ sensor synaptotagmin do not change in the KO (Supplementary Fig. 6b), we investigated Ca^2+^ entry using the low affinity calcium indicator dye Fluo5F-AM. Identifying presynaptic boutons with the red-shifted styryl dye FM4-64, we first stimulated at 10 Hz for 60 s and observed a substantially smaller increase in bouton Ca^2+^ in NHE9 KO neurons relative to WT (Fig. 6a). Surprisingly, we also observed a significant slowing of fluorescence decay post-stimulus (Fig. 6a), suggesting that the observed reduction in peak Ca^2+^ may underestimate the full extent of the defect in entry since it is compensated in part by impaired channel inactivation or reduced clearance. To focus more specifically on Ca^2+^ entry rather than Ca^2+^ accumulation in response to trains, we used Fluo5F to image boutons in response to single action potentials. The NHE9 KO shows a decrease in peak Ca^2+^ response to one action potential, but in contrast to the trains, no change in decay (Fig. 6b). The differential effect of the KO on Ca^2+^ decay after trains raised the possibility that this phenomenon might contribute to the increased paired-pulse ratio (Fig. 4b, c). However, the peak Ca^2+^ response to paired-pulse stimulation showed the same reduction in NHE9 KO neurons as in response to a single action potential, no change in the rate of decay, and normal facilitation of response to the second stimulus relative to the first (Fig. 6c, Supplementary Fig. 7a). Further, resting cytosolic Ca^2+^ measured with the high-affinity ratiometric indicator Fura-2AM showed no change in the NHE9 KO (Supplementary Fig. 7b). Thus, loss of NHE9 specifically impairs presynaptic Ca^2+^ entry.

A number of voltage-gated Ca^2+^ channels with distinct physiological properties mediate the Ca^2+^ entry required for neurotransmitter release. To identify the channels affected by loss of NHE9, we used a combination of snail toxins that inhibit specific Ca^2+^ channel isoforms. Imaging the response to a single action potential with Fluo5F, loss of NHE9 again impairs the Ca^2+^ signal (Fig. 6d, right panel). ω-Agatoxin TK, which specifically blocks P-type and Q-type Ca^2+^ channels, inhibited Ca^2+^ influx into the boutons of both WT and KO neurons, reducing but not eliminating the difference between them. The addition of ω-conotoxin GVIA, inhibiting N-type channels, further reduced the Ca^2+^ signal, effectively eliminating the difference between genotypes (Fig. 6d, right panel). Thus, loss of NHE9 selectively impairs Ca^2+^ entry through P-/Q- and N-type channels (Fig. 6d, left panel).

To determine how loss of NHE9 affects presynaptic Ca^2+^ channels, we first examined the α_1_A subunit, which forms the pore of P-type and Q-type channels^46^. Western analysis of hippocampal extracts shows no difference in α_1_A expression between WT and NHE9 KO mice (Supplementary Fig. 7c). Similarly, loss of NHE9 has no effect on α_1_A immunofluorescence intensity or synaptic localization in hippocampal cultures. Changes in α_1_A thus seem unlikely to account for the defective neurotransmitter release in NHE9 KO mice. Since auxiliary subunit α_2_δ can control the activity of Ca^2+^ channels^47,48^, we then examined isoform α_2_δ-1. Similar to the pore-forming subunit α_1_A, α_2_δ-1 shows no change in amount by western analysis of hippocampal extracts (Supplementary Fig. 7c).

Since a change in membrane trafficking might not involve a change in protein amount or even steady-state localization by immunofluorescence, we also examined the cell surface expression of α_1_A and α_2_δ-1 subunits by biotinylation. However, the results show no effect of the NHE9 KO on the total or surface fraction of α_1_A or α_2_δ, the glutamate transporter EAAT1, the GPI-anchored prion protein PrPc, AMPA, or NMDA receptors (Supplementary Fig. 7d). The changes in glutamate response must therefore reflect either altered synaptic localization or a difference in association with auxiliary proteins.

### Bafilomycin rescues synaptic vesicle exocytosis

To determine whether the defect in exocytosis might reverse acutely with synaptic vesicle alkalinization, we used the H^+^-ATPase inhibitor bafilomycin. However, H^+^ pump inhibition eliminates the changes in pH required to monitor exocytosis with pHluorin-based reporters and to fill synaptic vesicles for postsynaptic recording of transmitter release, requiring an alternative, pH-independent method to assess the exocytosis of recycled vesicles. We therefore relied on the pH-insensitive styryl dye FM2-10. Loaded into recycling synaptic vesicles during and for a short time after stimulation, the dye is destained by subsequent stimulation, providing another estimate of synaptic vesicle exocytosis. Interestingly, previous work has shown that bafilomycin loaded into recycling synaptic vesicles with the FM dye does not affect subsequent FM dye destaining, suggesting that acidification has no effect on synaptic vesicle exocytosis^49^. On the other hand, a recent study has shown that vesicle filling can influence synaptic vesicle exocytosis^50^, implying a role for the H^+^ electrochemical driving force.

**Fig. 7:**
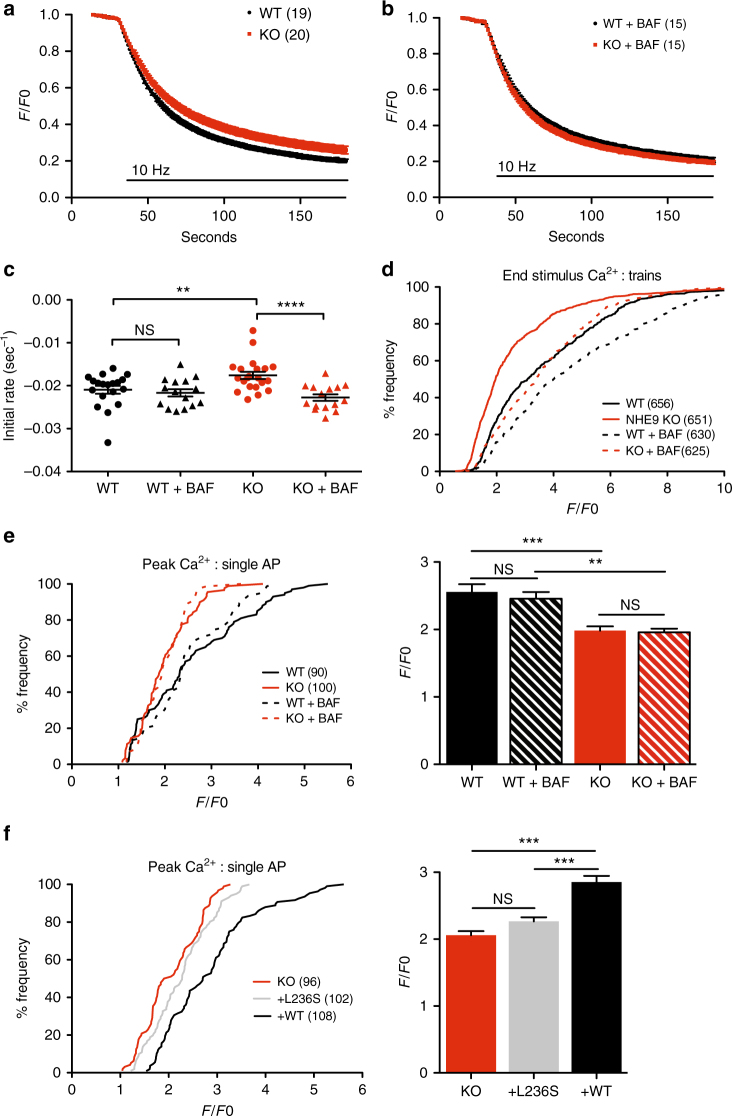
Intracellular alkalization rescues Ca^2+^ entry through two distinct mechanisms. **a** FM2-10 destaining confirms the defect in synaptic vesicle exocytosis. Hippocampal neurons were loaded with FM2-10 in bicarbonate-buffered media by stimulation at 10 Hz for 120 s, allowed to recover for 10 min, then stimulated again at 10 Hz for 150 s to unload the FM dye. **b** Neurons from the NHE9 KO loaded simultaneously with bafilomycin as well as FM2-10 do not differ from WT neurons in the rate of FM dye destaining in response to 10 Hz stimulation. **c** The initial rate of destaining shows that bafilomycin rescues the exocytosis defect of NHE9 KO neurons but does not affect the destaining of WT cells. **p < 0.01, ****p < 0.0001 by one-way ANOVA with Bonferroni comparison. WT, *n* = 19/15 coverslips/3 cultures; KO, *n* = 20/15 coverslips/3 cultures. **d** Cumulative frequency distribution of Ca^2+^ in boutons imaged using Fluo5F shows that bafilomycin loaded into the recycling synaptic vesicles substantially augments Ca^2+^ entry in both WT and KO neurons stimulated at 10 Hz. *n* = total number of boutons analyzed from 22 coverslips. WT, *n* = 22/21 coverslips/3 cultures; KO, *n* = 21/21 coverslips/3 cultures genotype/condition. **e** Cumulative frequency distribution (left) and mean peak (right) of the Ca^2+^ response to single action potentials before and after synaptic vesicle loading with bafilomycin. Bafilomycin does not change peak Ca^2+^ influx. **p < 0.01, ***p < 0.001 by one-way ANOVA with Bonferroni. *n* = 90–100 responses averaged from 30 boutons/coverslip across 14–15 coverslips and 3 cultures/genotype. **f** Cumulative frequency distribution (left) and mean peak (right) of the Ca^2+^ response to single action potentials in KO neurons transfected for 7 days with empty vector control (KO), NHE9 L236S mutant (+L236S) or wild-type NHE9 (+WT) plasmids. ***p < 0.001 by one-way ANOVA with Bonferroni. *n* = 96–108 responses averaged from 3 neurons/coverslip across 6 coverslips and 3 cultures/genotype. Data in **c**, **e**, and **f** indicate mean ± s.e.m. NS not significant

Using FM2-10, we found that the loss of NHE9 impairs synaptic vesicle exocytosis (Fig. 7a, c), consistent with the postsynaptic recording and VGLUT1-pHluorin imaging. We then used bafilomycin to inactivate the H^+^ pump acutely in the recycling synaptic vesicles loaded with styryl dye (Supplementary Fig. 8a). Bafilomycin eliminates the difference between NHE9 KO and WT in the rate of FM dye destaining, but has no effect on the WT (Fig. 7b, c), consistent with previous work^49^. Thus, excessive acidification due to the loss of NHE9 can impair synaptic vesicle exocytosis, and reverses acutely by simple dissipation of the H^+^ electrochemical gradient in recycling synaptic vesicles.

If acute bafilomycin treatment rescues the defect in exocytosis due to loss of NHE9, then it should also rescue the defect in calcium entry. After loading the bafilomycin into recycling vesicles, we thus imaged Ca^2+^ with Fluo5F. Preventing the acidification of recycling vesicles with bafilomycin strongly potentiates Ca^2+^ influx in response to trains (Fig. 7d). Indeed, previous work has shown that the high local concentration of protons released during synaptic vesicle exocytosis directly inhibits presynaptic Ca^2+^ channels^51^ and this phenomenon presumably accounts for the potentiation by bafilomycin. However, the difference in Ca^2+^ entry between WT and KO persists despite collapse of the proton gradient with bafilomycin. Thus, loss of NHE9 impairs presynaptic Ca^2+^ entry through a distinct mechanism that cannot be reversed acutely simply by inhibiting vesicle acidification. Consistent with this, preloaded bafilomycin does not increase the Ca^2+^ entry produced by a single action potential (Fig. 7e), indicating that bafilomycin specifically affects trains, which produce a greater increase in synaptic H^+^ than single stimuli. Interestingly, bafilomycin also shortens the time constant for Ca^2+^ decay in response to both trains and single action potentials, and eliminates the difference between genotypes for the response to trains (Supplementary Fig. 8b, c). An acute increase of endolysosomal pH thus rescues presynaptic Ca^2+^ entry and clearance but without affecting the primary defect in Ca^2+^ entry due to loss of NHE9. The defect in Ca^2+^ entry does not therefore reflect a change in the accumulation of extracellular H^+^ that occurs with stimulation.

It is possible that the defect in presynaptic Ca^2+^ entry reflects the loss of NHE9 protein rather than a loss of catalytic activity. To test this possibility, we transfected NHE9 KO neurons with either WT mouse NHE9 or a mutant associated with autism (L236S) that lacks catalytic activity but expresses at WT levels^16^. Co-transfection with VAMP2-pHluorin confirms that WT but not L236S NHE9 rescues the pH of axonal membranes (Supplementary Fig. 8d). We then investigated the effect of these two constructs on Ca^2+^ influx at presynaptic boutons of NHE9 KO neurons identified using co-transfected synaptophysin-mCherry. Expression of WT but not catalytically inactive L236S NHE9 rescues the defect in Ca^2+^ influx (Fig. 7f). A long-term increase of endosome pH thus appears required to reverse the defect in Ca^2+^ entry.

## Discussion

In contrast to the severe phenotype produced by loss of NHE6 in humans and mice^15,17^, loss of NHE9 has more selective effects on behavior, with no premature lethality, motor defects, or anxiety. Humans with mutations in NHE9 have epilepsy^22^ and the KO mice have high-voltage spike activity by EEG but very few obvious seizures, similar to another mouse model^28^.

The NHE9 cKO exhibits behavioral defects specifically related to social interactions. As juveniles, they solicit less play, and show less affiliative and investigative behavior. The recent analysis of another NHE9 KO line has also indicated reduced ultrasonic vocalizations in pups^28^. As adults, NHE9 cKO mice do not differ from WT in sociability, but fail to habituate on repeat exposure to the same animal, indicating a defect in social recognition. Social recognition has recently been suggested to depend on the hippocampus^27^, which expresses relatively high levels of NHE9.

The NHE9 cKO shows additional behavior associated with ASD. They bury more marbles than WT mice, suggesting obsessive or repetitive behavior. The NHE9 cKO also fails to distinguish socially relevant odors even though they have no apparent defect in basic olfaction, and this may contribute to the defect in social recognition. We do not identify the anatomic locus of this defect, but the olfactory bulb projects to both entorhinal cortex and hippocampus. Interestingly, recent work has shown that children with ASD sniff equally regardless of odor valence, and aberrant sniffing behavior correlates strongly with ASD severity in humans^52^. Surprisingly, heterozygous NHE9 mice also exhibit multiple defects in juvenile play. Loss of NHE9 in mice, either conditionally in the nervous system or as a full KO, thus reproduces much of the repetitive behavior and impaired communication associated with defective sensory processing and social impairment in ASD.

Although yeast *Nhx1* contributes to the regulation of cytosolic pH^11^, loss of NHE9 has no effect on cytosolic pH in neurons. In contrast, loss of NHE9 lowers the pH of dendritic and presynaptic endosomes. Despite a role for NHE7 in acidification^39^, NHE9 thus appears to catalyze endosomal alkalinization. NHE6 has a similar role in regulation of the endosome H^+^ electrochemical gradient. Loss of NHE6 also results in the hyperacidification of endosomes^15^, indicating an analogous role as H^+^ leak. However, the two isoforms are not redundant since loss of either produces a phenotype. This may reflect differences in subcellular localization, as originally suggested^36^. However, NHE6 localizes to endosomes^15,53,54^, much like NHE9. In the axon, NHE6 localizes to synaptic vesicles by proteomics^55^ which our lab has confirmed by gradient fractionation. Although we cannot detect endogenous NHE9, expression of epitope-tagged transporter in the NHE9 KO suggests only partial localization of the axonal protein to synaptic vesicles. A substantial fraction of NHE9 localizes to axonal puncta not labeling for synaptic vesicle proteins, and the primary effect of the KO on pH of more alkaline vesicles (rather than the more acidic recycling synaptic vesicles) further supports a role in axonal endosomes. The isoforms may thus vary in their abundance on different endosomal membranes, with each contributing in a quantitative way to lumenal pH. The more severe phenotype of NHE6 inactivation may indeed simply reflect the greater abundance of this isoform.

In light of previous work suggesting a role for NHEs in vesicle filling^14^, we were surprised to find no change in the vesicular transport of either GABA or glutamate, with or without K^+^ to activate the associated NHE. Indeed, recent work using the functional reconstitution of recombinant protein has suggested that the VGLUTs may themselves confer synaptic vesicle NHE activity^56^.

Loss of NHE9 nonetheless has a major effect on basal synaptic transmission. The paired-pulse ratio increases in both field and whole cell recordings, indicating reduced release probability^57^. Direct imaging of synaptic vesicle exocytosis using a pHluorin-based reporter and an FM dye confirm the defect inferred from postsynaptic recording. We have thus observed the same phenotype for release in both dissociated culture from newborns and acute slices from young adults. NHE9 expression increases during development but the effect of the KO on release remains remarkably consistent over this time.

In contrast to the reduced probability of evoked release, the frequency of spontaneous release increases. This does not appear to reflect an increase in excitatory synapse number, and the reduced AMPA/NMDA ratio does not suggest an increase in functional synapses. We also find no change in the total amount of AMPA or NMDA receptors on the plasma membrane, suggesting that the reduction in AMPA/NMDA ratio may reflect a redistribution of cell surface receptors away from the synapse. Taken together, the electrophysiology suggests that loss of NHE9 has opposing effects on evoked and spontaneous release, consistent with recent work suggesting that the two forms of release may derive from different populations of synaptic vesicles^37,58–60^. Further, the single cell inactivation experiments demonstrate that the postsynaptic and probably the presynaptic effects of NHE9 inactivation are cell autonomous.

Together with the disturbance in excitatory neurotransmission, the reduced mIPSC amplitude suggests an explanation for the seizures observed in patients with mutations in NHE9^22^. Although we detected very few convulsions in the KO mice, the high-voltage activity on EEG has been suggested as a precursor to the spike-and-wave of absence epilepsy^61^. In the NHE9 KO, the frequency (3–4 Hz) is lower and duration (minutes) is longer than previously reported, implicating a distinct physiological mechanism, but the prolonged, associated immobility is characteristic of high-voltage spike activity and suggestive of an abnormal cortical state^33^.

How does loss of NHE9 influence synaptic vesicle exocytosis? Previous work has indicated a role for ΔpH in homotypic fusion of the yeast vacuole, but in that case it promotes the pairing of SNARE proteins from opposing membranes required for membrane fusion^62^. A subunit of the vacuolar H^+^-ATPase itself has also been implicated in membrane and even synaptic vesicle fusion, but this function appears independent of its role establishing the H^+^ electrochemical gradient^63,64^. We now find that loss of NHE9 impairs evoked neurotransmitter release by reducing presynaptic Ca^2+^ entry. The KO has no effect on readily releasable or recycling pool size, and direct measurement with Fluo5F shows less Ca^2+^ entry in response to either trains or a single action potential. Increasing external Ca^2+^ eliminates the difference from WT, further demonstrating that reduced Ca^2+^ entry accounts for the defect in release. By imaging presynaptic Ca^2+^ entry, we find that loss of NHE9 selectively affects N-type and P-/Q-type channels. The pore-forming α_1_A subunit associated with P-/Q-type channels shows no change in expression or cell surface localization.

How does a disturbance in the H^+^ electrochemical gradient affect presynaptic Ca^2+^ entry? In adrenal medullary cells, dissipation of the chromaffin granule pH gradient can stimulate release by increasing cytosolic Ca^2+^, presumably through effects on intracellular Ca^2+^ stores^65–67^. On the other hand, previous work using FM dyes has suggested that dissipation of the H^+^ electrochemical gradient does not influence synaptic vesicle exocytosis^49,68^. Indeed, we also find that the H^+^ pump inhibitor bafilomycin loaded into recycling synaptic vesicles does not influence subsequent FM dye destaining in WT cells. However, preloaded bafilomycin does rescue the defective response to stimulus trains in the absence of NHE9. Direct imaging of presynaptic Ca^2+^ also shows potentiation by bafilomycin, but only in response to trains, not to single action potentials. Indeed, previous work has shown that H^+^ released by synaptic vesicle exocytosis can inhibit presynaptic Ca^2+^ channels^51,69^, and this would account for the selective effect of bafilomycin on Ca^2+^ entry with trains rather than single action potentials. Loss of NHE9 might exacerbate this inhibition, but preloaded bafilomycin increases Ca^2+^ entry in WT as well as KO, and the difference between genotypes remains in response to trains as well as single action potentials. Thus, acute treatment with bafilomycin potently increases presynaptic Ca^2+^ channel function, but does not rescue the primary defect in presynaptic Ca^2+^ entry due to loss of NHE9 which must therefore involve a distinct mechanism. In contrast, expression of NHE9 rescues the defect in presynaptic Ca^2+^ entry, suggesting a more long-term effect of the exchanger.

Consistent with a role for vesicle pH in the phenotype, we also found that rescue of the defect in presynaptic Ca^2+^ entry requires the catalytic activity of NHE9. WT but not catalytically inactive L236S NHE9 rescues the hyperacidication of axonal vesicles as well as Ca^2+^ influx in response to single action potentials. This result does not distinguish between different mechanisms for the effect on Ca^2+^ entry, but indicates that lumenal pH is the crucial factor.

## Methods

### Mouse breeding and genotyping

To produce a cKO of NHE9, JM8A3.N1 embryonic stem (ES) cells (derived from C57Bl/6) were transfected with the targeting vector described in Figure 1A, selected in G418 and screened by long-range PCR for homologous recombination at a single NHE9 allele (Knockout Mouse Project). To generate chimeras, the appropriately targeted ES cells were then injected into C57Bl/6 recipient blastocysts (University of California at Davis Mouse Biology Program). The resulting chimeras were bred to C57BL/6 mice to produce heterozygotes, and the heterozygotes bred to mice expressing FLPe under the control of the β-actin promoter (Tg(ACTFLPe)9205Dym) to remove the lacZ reporter and selectable marker^70^. To inactivate NHE9 in the nervous system, inbred mice homozygous for loxP sites flanking exon 5 (fl/fl) were crossed with heterozygous (fl/+) mice expressing cre under the control of the nestin promoter (Tg(Nes-cre)1Kln)^71^. Since nestin-cre inactivates NHE9 at low frequency in gametes, we also used these animals to produce unconditional heterozygotes (fl/− without cre) as well as cKO animals (fl/−; cre/+). We also used mice fully recombined at the NHE9 locus (−/−) for hippocampal primary culture as well as acute hippocampal slice recording. It is important to note that the ES cells and all subsequent mice used to mate with the NHE9 KO animals were on a C57Bl/6 background, eliminating the requirement for back-crossing.

Genotyping was performed by PCR using the primers indicated (all from 5′ to 3′) (Supplementary Fig. 1a). For WT NHE9, we used the 5′ homology arm primer Slc9a9-F: GGCCAGACTTTGGTTGGTCATTCC (#1), and a primer from the sequences deleted by homologous recombination (WT-R only): GGCTATGTACCATGCATATCCTTTTGG (#2). To detect the original insertion event, we used a primer through the most 3′ loxP site (CSD-loxP-F): GAGATGGCGCAACGCAATTAATG (#3) and the 3′ homology arm primer Slc9a9-R: ACCCCGATTCTGATTAAGCCTCTAGC (#4). To detect the presence of the lacZ reporter, we used the primer pair LacZ-F: GTGCGGATTGAAAATGGTCT (#5) and LacZ-R: TATTGGCTTCATCCACCACA (#6). To detect the neomycin-resistance marker, we used the primer pair NEO-F: GCCATCACGAGATTTCGATT (#7) and Slc9a9 Exon 5-R: GCACCATGGCTTTCACAAA (#8). To detect the nestin-cre transgene, we used the primer pair nestin-CRE-F: AATGCTTCTGTCCGTTTGC and nestin-CRE-R: TAGCGCCGTAAATCAATCG. To detect deletion of exon 5, we used a combination of Slc9a9-F with either Slc9a9-R or Slc9a9 Exon 5-R.

To confirm targeting of the conditional allele, mouse tail DNA was subjected to Southern analysis. Briefly, genomic DNA was digested with restriction enzymes *Spe*I and/or *Sbf*I, which cut the Slc9a9 locus upstream of the 5′ homology arm and downstream of the 3′ homology arms, respectively. The DNA was blotted onto Zeta-probe GT (Bio-Rad) cationized nylon membranes and hybridized with sequences outside the homology arms that were amplified by PCR and labeled using Rediprime (Amersham).

### NHE9 mRNA expression

At each time point, the brains of three C57Bl/6 mice were rapidly frozen in liquid nitrogen and total RNA extracted using Trizol reagent. Purified RNA (1 µg) was converted into cDNA with oligodT primers, and the primers were tested for efficiency using a 10-fold dilution of cDNA. To amplify Slc9a9 sequences by PCR, we used the following primer pairs: exon 5–6 forward primer (TTGTGAAAGCCATGGTGCAT) and reverse primer (GACTATGGCCACCGCATCAT); ACT forward (CGTCGACAACGGCTCCGGCA) and reverse primers (CCCATTCCCACCATCACACCCTGGT); exon 2–6 forward primer (CCAGAGAGAGATCAACCAGCA) and reverse primer (GAGGACTATGGCCACCGCATCAT). qRT-PCR was performed in triplicate using SYBRgreen and the mean fluorescence used to calculate the delta-delta cycle threshold (CT) fold change for each transcript^72^.

### Mice

The subject mice used for all of the behavioral tests on adults were 3–12-month-old male KO (−/−), cKO (fl/−;cre/+) and HET (fl/−) mice with WT (fl/fl) littermates as controls. Mice were housed with enrichment, in the same cohort of 4–5 animals/cage for the entire testing period. C57Bl/6 males 4–8 weeks old were used as control novel mice. Before and after each test, all equipment was cleaned thoroughly with soap and water, and then with ethanol. Unless otherwise stated, all experiments were performed during the light cycle from 9 a.m. to 6 p.m., and at the same time frame for each experiment. Only one test/animal was performed each day. All behavioral studies were performed and analyzed blind to genotype. All experiments with animals were performed according to the National Institutes of Health Guide for Care and Use of Laboratory Animals and were approved by the University of California San Francisco Institutional Animal Care and Use Committee.

### Behavioral assays

Open field activity was assessed as previously described^73^. Mice were habituated first to the testing room for 30 min, then habituated to the chamber for 30 min before testing. The chamber was a clean plexiglass rat cage (44 × 20 × 30 cm^3^) with opaque sides. Video was recorded for 30 min of open field exploration. At the end of the trial, a wire cup was placed in the center of the cage for the novel object exploration, and video was recorded for an additional 30 min. Automated video analysis was performed with Ethiovision XT tracking software (Noldus Technology) by setting the arena space (44 × 20 × 30 cm^3^), the center space (14 × 12 cm^2^ centered), tracking the mouse, and recording the total distance moved and total time spent in each arena. All data are presented as mean ± s.e.m. and analyzed by one-way analysis of variance (ANOVA) with Bonferroni post hoc comparison.

For elevated plus maze, mice were habituated to the test room for 60 min and the test performed essentially as described^74^. The maze platform was elevated 50 cm off the ground. Comprised of two closed arms (10 × 50 cm^2^), it was surrounded by 40 cm high non-transparent walls and two open arms (10 × 50 cm^2^). During the 5 min trial, times spent in closed, center, and open arms, and as well as total number of entries/quadrant were tallied in real time by investigator blind to genotype. All data are presented as mean ± s.e.m. and analyzed by two-way ANOVA.

For rotarod assay, mice were habituated to the test room for 30 min and placed walking forward in their respective lane on the rotarod with the machine rotating at 4 r.p.m. With all animals on the rotarod, acceleration increased at a steady rate from 4 to 40 r.p.m. over 300 s. The latency to fall off was recorded for each animal. The trial was repeated three times, with 15 min intertrial intervals (ITIs). All the data are presented as mean ± s.e.m. and analyzed by two-way ANOVA.

Juvenile play was assessed as previously described^73^. Briefly, 3-week-old mice were habituated to the testing environment one day before the test for 1 h in standard individual mouse cages with food and water. Every animal was weighed so that play pairs could be weight matched, and half the animals received a mark on their tails with a silver sharpie so that the individuals in a pair could be distinguished more easily during analysis. On the day of testing, mice were habituated to the room for 1 h and then to the test cage (standard clean mouse cage) for 10 min. Paired mice not housed together (novel mice) were allowed to interact for 30 min while videotaped. The behaviors scored included investigative (sniffing or following the other mouse), affiliative (allogrooming, close physical contact), and play soliciting (crawling over/under, touching while pushing past, lodging under)^73^. For each animal, the time spent engaged in specific activities was scored by experimenter blind to genotype for the entire 30 min recording. Data are presented as mean ± s.e.m. and analyzed using one-way ANOVA with Bonferroni post hoc comparison.

The three chamber sociability test was performed as previously described^75,76^. Male C57Bl/6 mice housed separately from the subject animals were habituated to the testing room for 1 h, then placed under a wire cup for 30 min before use as novel test animals. The chamber used for testing was constructed of clear plexiglass with overall dimensions and left/center/right chamber dimensions as previously described, but with no automated doors or infrared beams to detect movement. This chamber was encased in a larger opaque plastic box with a hole at the top for a video camera. Subject mice were habituated to the testing room for 1 h, then placed into the center chamber with both doors closed for 5 min. The doors were then opened, and the mice were allowed to explore all three chambers for 10 min. Analysis of the time spent in each chamber during this phase enabled us to conclude that there was no chamber bias. Next, an upside-down novel wire cup was placed in one chamber, a wire cup enclosing a novel mouse in the other chamber, and the subject mouse was allowed to explore all three chambers for 10 min while recorded. For the final 10 min of recording, the empty wire cup was replaced with a different wire cup enclosing a new novel test mouse and this phase recorded for an additional 10 min. Movement, time spent per chamber and time spent investigating the wire cups was automatically measured using Ethiovision XT tracking software (Noldus Technology). All data are presented as mean ± s.e.m. and analyzed by two-way ANOVA with Bonferroni post hoc comparison.

The social recognition test was performed as previously described^27^. Briefly, subject and novel test C57Bl/6 mice were habituated to the testing room for 60 min. The subject mouse was then habituated to the test chamber (a clean standard mouse cage) for 5 min before adding the novel test mouse (M1) for 5 min. After an additional 60 min, M1 was reintroduced for 5 min, and at 120 min, a new novel mouse (M2) was added for 5 min. Total time spent by the subject mouse investigating (sniffing, allogrooming, following within 2 cm) the novel mice was determined in real time blind to genotype. Results are expressed as mean ± s.e.m. and analyzed by one-way ANOVA with Bonferroni post hoc comparison.

Before the marble burying assay, mice were habituated to the testing room for 60 min before the actual test, which was performed as previously described^29,76^. The test cage was a standard mouse cage containing 4.5 cm sani-chip bedding with 20 marbles (blue, glass, 1.5 cm diameter) placed in a regular array (4 × 5) across the top of the bedding. Subject mice were placed in the test cage and left for 30 min. The animals were removed at the end of this time and the total number of marbles buried at least 2/3 in the bedding recorded for each cage. Average marbles buried per genotype were statistically compared by one-way ANOVA.

For odorant investigation test, mice were habituated to the testing room for 30 min before testing and the test performed essentially as previously described ^76,77^. The test cage was a standard mouse cage with a small hole in the lid for a scent applicator. Cotton tip applicators were soaked in odorants in this order: water, almond extract (frontier 1:100), banana extract (frontier 1:100), or swiped across the bottom of a dirty cage (unchanged for a week) from novel males or novel females (both C57Bl/6), bobcat urine (The PeeMart, 1:100). The test mouse was habituated to the test cage for 2 min with an untreated cotton swab suspended from the lid. Odorant swabs were individually presented through the lid for 2 min each, and then removed for an ITI of 1 min before the next odorant was presented. The cumulative time spent sniffing the cotton swab was recorded per exposure. Data are presented as mean ± s.e.m. and analyzed by two-way ANOVA with Bonferroni post hoc comparison.

The buried cookie assay was peformed as previously described^78^, mice were habituated to novel food (one ~1.5 g teddy graham cookie per mouse per day) for 3 days before testing. The evening before the test, all food was removed from the home cage. The next morning, mice were habituated to the test room for 60 min. A single cookie was hidden in a cage buried under 3 cm sani-chip bedding. Mice were habituated to sani-chip bedding in a separate cage for 5 min before entering the test cage. Once in the test cage with the hidden cookie, the time required to find the buried cookie was recorded. The average delay was calculated per genotype and the results analyzed by one-way ANOVA.

For hot plate test, mice were habituated to the room for 30 min before testing. The surface of the hot plate was maintained at 55^o^C, the mouse placed into a 500 ml glass beaker and the latency to show a nociceptive response via hind paw lick, hind paw flick, or jumping was recorded. The mouse was removed as soon as the animal showed any sign of nociceptive response. The average latency was calculated per genotype and the results analyzed by one-way ANOVA.

### Electroencephalogram

For the EEG, intracranial electrodes were implanted bilaterally at the medial prefrontal cortex (mPFC) of 3-month-old NHE9 KO and C57Bl6/N male mice and 3–5 days later the differential EEG was recorded. Specifically, after anesthesia with isofluorane to an areflexive state, conductive stainless steel screws (the recording electrodes) were implanted intracranially at the following coordinates (in millimeters relative to bregma): mPFC: +1.7 anterior–posterior (AP), 0.6 mediolateral (ML), −2 dorsal–ventral (DV); temporal cortex: −2.5 AP, 3.5 ML, −2 DV. A screw serving as ground electrode was also implanted at −3 AP, −3 ML, and −2 DV. All screws were attached to a head mount using conductive wire, the head mount secured with dental cement, and the mice allowed to recover for 3–5 days before recording. The EEG was recorded as the difference between left and right prefrontal electrodes at 2 kHz using a time-locked video EEG monitoring system (Pinnacle Technology), and the spectrograms computed using the *spectrogram* function in Matlab (Mathworks, Natick, MA, USA).

### Neuronal culture

Hippocampal neurons were isolated from newborn mouse pups following guidelines approved by the UCSF IACUC. Hippocampi were digested for 20 min in Hanks' buffered saline + 0.36% glucose (HBS+) containing 0.00025% trypsin, washed three times with HBS+, and triturated 30 times in 2.25 ml MEM containing 21 mM glucose, 5% fetal bovine serum, 2% B27 and 1 × Glutamax (Serum Neuronal Media, SNM). Coverslips pretreated with l-polylysine (PLL) were rinsed in water and dried before plating neurons. For live cell imaging experiments, neurons in SNM were plated into 10 mm cloning rings secured onto 25 mm coverslips at a density of 30–35,000/79 mm^2^. For immunofluorescence, neurons were plated onto 12 mm coverslips at a density of 45,000/113 mm^2^. For biochemical experiments, neurons were plated directly onto PLL-coated wells of a 12-well plate at a density of 150,000/380 mm^2^. The next day, 50% of the SNM media was removed and 3 volumes Neurobasal Media (NBM) 1 days in vitro (DIV) (NBM with 2% B27, 1.3 × Glutamax) was added. At DIV7, 50% of the media were removed and one volume NBM DIV7 (1 part SNM: 3 part NBM DIV1, 4 µM AraC) added. Neurons were transfected using calcium phosphate at DIV6, resulting in transfection efficiencies ~15%. For biochemical experiments, neurons were infected with lentiviral supernatant at DIV7 resulting in 100% transduction efficiency. For the ss-flag-DOR lysosomal degradation assay, DOR agonist DADLE was applied at a final concentration of 10 µM directly into culture media for the times indicated.

### Immunofluorescence

Cultured neurons at DIV14 were fixed in phosphate-buffered saline (PBS) containing 4% paraformaldehyde (and 4% sucrose in the case of Ca^2+^ channel labeling) for 20 min at 27 °C. The cells were then washed three times for 5 min each in PBS, and permeabilized/blocked in PBS containing either 0.01% saponin, 2% normal goat serum (NGS) for 60 min at 27 °C or 0.1% Triton X-100, 10% NGS (3% NGS for antibody incubation) for 30 min (in the case of Ca^2+^ channels). Incubation in primary antibody was performed in the same buffer for 60–120 min, followed by washing and incubation in secondary antibody for 60 min. Coverslips were mounted with fluoromount and images acquired using a Zeiss LSM 510 confocal scanning microscope. We used the following antibodies and titers for immunostaining: TfR (Invitrogen, 1:500), VGLUT1 (Chemicon, 1:5000), VGAT (Synaptic Systems, 1:1000), GluR2 (Millipore, 1:1000), LAMP1 (Developmental Studies Hybridoma Bank, 1:400), NHE6 (gift of J. Orlowski, adsorbed), GFAP (Zymed, 1:1000), Ca^2+^ channel subunit α-1A (Synaptic Systems, 1:1000), and α2-δ1 (Alomone, 1:100). Cy3-conjugated and Cy5-conjugated secondary antibodies (Jackson Immuno Research) and Alexa 488-, 555-, or 649-conjugated secondary antibodies (Invitrogen) were used at a dilution of 1:1000.

The cross-correlation between NHE9-HA^+^ and TfR^+^ endosomes or VGLUT1^+^ boutons was determined using two methods. In both cases, the images were masked to visualize only signal present within the GFP^+^ neurons. To determine cross-correlation, both channels were thresholded and the ImageJ particle analysis plugin (granulometric filter) used to generate regions of interest (ROIs) for TfR and VGLUT1. The number of ROIs with average NHE9-HA signal >10 arbitrary fluorescence units per ROIs were then divided by the total number of ROIs. For the standard Manders cross-correlation, the ImageJ plugin JACoP was used to determine the overlap of TfR and VGLUT1 signal with NHE9-HA, again within the masked area.

### Live neuron imaging

Cultured neurons were imaged at DIV12–14 to measure both vesicular and cytosolic pH. For vesicular measurements, a pHluorin fusion to TfR and VAMP2, as well as mOrange2 fusion to VGLUT1 were respectively used as dendritic endosome, axonal endosome, and synaptic vesicle reporters. We used mOrange2 (p*K*a ~6.8) rather than the ecliptic pHluorin for the fusion to VGLUT1 because the pHluorin (p*K*a ~7.1) is completely quenched at the pH of synaptic vesicles (~5.8), making it difficult to detect any lower pH^79^. The pHluorin was imaged using 470/40 nm excitation and 525/50 nm emission filters, and mOrange2 using 545/30 nm excitation and 595/50 nm emission filters. Glutamate receptor antagonists 6-cyano-7 nitroquinoxaline-2,3-dione (CNQX, 10 μM) and d,l-2-amino-5-phosphonovaleric acid (APV, 50 μM) were included in the external solution for all imaging experiments. Lumenal pH (pHi) was measured as previously described^42^ assuming a p*K*a for pHluorin ~7.1 and mOrange2 ~6.8. Total pHluorin emission is a combination of surface and intravesicular fluorescence. Baseline fluorescence was obtained as an average of five frames collected at 0.2 Hz in Tyrode’s solution (mM: 100 NaCl, 2.5 KCl, 2 CaCl_2_, 2 MgCl_2_, 10 glucose, and 25 HEPES, pH 7.4). To determine the fraction quenched at low pH, we imaged the neurons during fast perfusion with Tyrode’s solution, pH 5.5, for pHluorin and pH 5.0 for mOrange2 (mM: 100 NaCl, 2.5 KCl, 2 MgCl_2_, 2 CaCl_2_, 10 glucose, 25 MES ((2-(*N*-morpholino)ethanesulfonic acid)), pH 5.5 (pHluorin), or 25 Homopipes pH 5.0 (mOrange2)), collecting five frames at 0.2 Hz and using the lowest fluorescence values for the acid quench. During the same acquisition period, intracellular compartments were alkalinized by fast perfusion with Tyrode’s solution, pH 7.4, containing 50 mM NH_4_Cl (mM: 50 NaCl, 2.5 KCl, 2 MgCl_2_, 2 CaCl_2_, 50 NH_4_Cl, 10 glucose, 25 HEPES, pH 7.4), thus revealing the lumenal pHluorin quenched by low pH. In principle, pHi was calculated as *f*(pHi) = (average baseline fluorescence – average pH 5.5 or pH 5.0 fluorescence)/peak fluorescence NH_4_Cl, and more precisely according to:

$$f\left( {\rm pHi} \right) = \frac{{\it{\epsilon }}}{{\left( 1 \right. - \phi \left( {\rm pHi} \right) + \propto \left( {\rm pHi} \right)(1 - {\it{\epsilon }})}},$$

where

$${\it{\epsilon }} = (F_0 - F_{\rm pH5.5})/F_0,$$

$$\phi \left( {\rm pHi} \right) = \frac{{1/(1 + 10^{{\rm p}K - 5.5})}}{{1/(1 + 10^{{\rm p}K - 5.5})}},$$

$$\alpha \left( {\rm pHi} \right) = \frac{{{\mathrm{\Delta }}[X]}}{{[X]_0}} = \frac{{\left( 1 \right./(1 + 10^{{\rm p}K - 7.4}) - 1/(1 + 10^{{\rm p}K - {\rm pHi}})}}{{1/(1 + 10^{{\rm p}K - {\rm pHi}})}}.$$

ROIs of 10 × 10 pixels were selected for TfR and 5 × 5 pixels for VAMP2. Background was determined by averaging 10 representative background ROIs of similar size without cells and the average subtracted from each experimental ROI of similar size. A minimum of 25 puncta per neuron from 3 to 5 neurons per coverslip were analyzed.

To measure cytosolic pH, cultured neurons were incubated with 1 µM BCECF-AM for 15 min in Tyrode’s solution, pH 7.4. Images of the soma and processes were acquired with 436/10 and 495/10 nm excitation and 530/35 nm emission filters. After imaging steady-state cytosolic pH, neurons were perfused with calibration solution containing 50 µM nigericin and (in mM) 100 KCl, 20 NaCl, 2.5 MgCl_2_, 25 HEPES (pH 8, 7.5, 7), or MES (pH 6.5, 6, 5.5). The ratio of fluorescence produced at the two excitation wavelengths for each pH was then used to produce a calibration curve, and this curve used to calculate the pH for each ROI. The difference between means for each genotype was assessed for significance using the Student’s *t* test.

To image the cycling of synaptic vesicles, cultured neurons were used at DIV14–17 as previously described^37^. VGLUT1-pHluorin with a C-terminal cytosolic mCherry tag was imaged with 470/40 nm excitation and 525/50 nm emission filters for the pHluorin, and with 572/30 nm excitation and 632/60 nm emission filters for mCherry. mCherry images were collected once each at the start and end of acquisition, and used to identify transfected boutons in DIV14 neurons. PHluorin images were acquired at 2 Hz, and the fluorescence normalized to total intracellular fluorescence (in NH_4_Cl) after subtracting the initial fluorescence, or *F*_NH4Cl_ – *F*_initial_. Background fluorescence in each frame was determined by translating 5 × 5 pixel ROIs 10 pixels into an area of representative, adjacent background, and subtracted from the fluorescence of each puncta. Endocytosis kinetics at the end of 10 Hz stimulation were determined by fitting the data to single exponential decay using PRISM (GraphPad). To calculate exocytosis rate and total recycling pool size, DIV14 neurons were stimulated at 10 Hz for 150 s in 600 nM bafilomycin. The average initial rate of exocytosis was determined for each neuron, and the average for each genotype compared using the Student’s *t* test. Recycling pool size was determined from the plateau reached during stimulation in bafilomycin and expressed as a fraction of F_NH4Cl_. The effect of genotype was assessed using the Student’s *t* test. Imaging DIV17 neurons at 1 Hz, the RRP size was measured as the peak response to 40 action potentials delivered at 20 Hz for 2 s, and the mean for each genotype compared by Student’s *t* test. Calcium sensitivity was similarly determined by stimulating DIV17 neurons on the same coverslip three times at 10 Hz each for 30 s with 10 min between stimuli, first with 2 mM, then 1 mM, and finally 4 mM Ca^2+^, followed by NH_4_Cl. The cells were imaged at 1 Hz, and the VGLUT1-pHluorin peak response to stimulation was normalized as above.

FM2-10 (50 μM) was loaded into synaptic vesicles of DIV14 hippocampal neurons by stimulating at 10 Hz for 120 s in culture media since it contains physiological bicarbonate buffer and only 10 mM HEPES. Bafilomycin (600 nM) was loaded simultaneously with FM2-10, followed by a rapid rinse with Tyrode’s solution, then Advasep (1 mM) in culture media for 2 min, and finally constant perfusion in Tyrode’s solution for 10 min before unloading FM2-10 by stimulation at 10 Hz for 150 s. Images were acquired at 1 Hz using 470/40 nm excitation and 525/50 nm emission filters. The time constant for destaining (tau) was calculated by nonlinear curve fitting of a plateau with one-phase decay using PRISM. The mean tau per genotype and experimental condition were compared by two-way ANOVA and Bonferroni post hoc comparison.

Fluo5F-AM (6 μM, Invitrogen) was loaded into DIV15–18 neurons for 15 min at 27 °C in Tyrode’s solution containing glutamate receptor antagonists. The cells were then washed for 30 min at 27 °C in fresh buffer. At the beginning of each experiment, boutons were loaded with FM4-64 (20 µM) by stimulation at 10 Hz. External FM dye was then removed by chelation in 1 mM Advasep (Biotium) for 2 min, followed by perfusion for 8 min in Tyrode’s solution. Images were acquired at the beginning of each experiment to identify boutons and at the end to verify stimulation-dependent unloading. Fluo5F was imaged with 470/40 nm excitation and 525/50 nm emission filters at 10 Hz for trains and 33.3 Hz for single action potentials. Background fluorescence was determined by averaging ten 5 × 5 pixel ROIs for each frame and subtracting that value from the fluorescence at each ROI. *F*_0_ was determined as the average value for 5 or 1 s before the onset of train or single action potential stimulation, respectively. ω-Agatoxin TK (300 nM, Alomone Labs) and ω-conotoxin GVIA (1 µM, Alomone Labs) were added to the neurons in Tyrode’s solution for 5 min before imaging. Drugs were then added sequentially so that each coverslip was imaged first without drugs, then with agatoxin, and finally with agatoxin and conotoxin. Δ*F*/*F*_0_ was used for the measurement of Ca^2+^ in the experiment so that a decrease in response would directly reflect the proportional contribution of each channel subset. Thirty to fifty boutons per coverslip were analyzed. For high-frequency stimulation, we determined the mean from the final 10 s. The fluorescence decay was fit to a single exponential using PRISM. The maximal response to a single action potential was used to determine peak Ca^2+^ influx and the decay constant determined by fitting the decay to a single exponential in PRISM.

DIV18–19 neurons were loaded with the high-affinity Ca^2+^ indicator Fura-2-AM (2 μM, Thermo Fisher) for 30 min at 37 °C, then perfused with Tyrode’s solution. To identify boutons, FM4-64 dye was loaded by stimulation as previously described, and neurons were allowed to rest for 10 min before imaging. Fura-2 was imaged ratiometrically at 1 Hz by excitation at 340/v2 and 380/v2 nm, with emission at 510/80 nm. Stimulation at 40 Hz for 10 s was used at the end of the imaging to verify neuronal health. Background fluorescence was determined by taking an average of ten 5 × 5 pixel ROIs for each frame and excitation wavelength, and subtracted from the fluorescence at an adjacent bouton.

### Hippocampal culture

After euthanasia, the hippocampi were dissected rapidly in cold HEPES-buffered saline, flash frozen in liquid nitrogen, and stored on dry ice until homogenization. The hippocampi were disrupted in 300 µl buffer containing 150 mM NaCl, 50 mM Tris, pH 7.5, 1 mM EGTA, 1% Triton X-100, 0.5% Na-deoxycholate and 1× Roche protease inhibitor cocktail using a Dounce homogenizer by hand for 60 s and by rotation at 4 °C for 30 min. The homogenate was then centrifuged at 1000 *g* for 10 min at 4 °C to sediment large debris. The supernatant was transferred to a new tube, total protein concentration determined by BCA, equal amounts of protein added to sodium dodecyl sulfate (SDS) loading buffer with 100 mM dithiothreitol (DTT), and 5% β-mercaptoethanol . The samples were incubated at 27 °C for 30 min before separation by electrophoresis through SDS-polyacrylamide (10–15%) and immunoblotting.

Extracts were prepared from primary dissociated cultures using lysis buffer (150 mM NaCl, 50 mM Tris, pH 7.5, 1% Triton X-100, 0.1% SDS, and 1× Roche protease inhibitor cocktail) at 100 µl/well. The plates were rocked at 27 °C for 5 min, then transferred to eppendorf tubes and solubilized by rocking at 4 °C for 60 min. Insoluble material was sedimented at 16,000 *g* for 10 min, protein concentration was determined by BCA to equalize loading, and aliquots were stored at −80 °C until western blotting as above.

### Cell surface biotinylation

Cell surface proteins of DIV18 cultured neurons were labeled by biotinylation and isolated by pull-down with Neutravidin. Briefly, culture media were removed and neurons washed four times with cold PBS (+Ca^2+^/Mg^2+^). The final wash was replaced with PBS containing 1 mg/ml NHS-SS-Biotin (Thermo) and the neurons incubated for 20 min at 4 °C with gentle rocking. NHS-SS-Biotin was quenched using 0.1 M glycine in PBS, then washed for 10 min with gentle rocking. Cells were immediately lysed in ice-cold RIPA buffer (150 mM NaCl, 50 mM Tris, pH 7.4, 1% Triton X-100, 0.1% SDS, 0.1% sodium deoxycholate) containing 1 × Roche protease inhibitor cocktail. After solubilization by rocking for 60 min at 4 °C, the lysates were sonicated in a water bath for 10 min and sedimented at 14K r.p.m. for 10 min in a microfuge to pellet large debris. The protein (75 µg) was then added to 50 µl Neutravidin Agarose resin beads (Thermo) in a total volume of 1 ml RIPA buffer and the mixture rotated overnight at 4 °C. The beads were then pelleted for 60 s at 5K r.p.m., washed with RIPA buffer, and the process repeated for a total of four times. After the final sedimentation, the beads were resuspended in 50 µl SDS gel loading buffer with 100 mM DTT and incubated at 37 °C for 60 min before electrophoresis through 4–20% Tris-glycine polyacrylamide and transferred to nitrocellulose membranes. The blots were probed with the antibodies described and the fluorescence quantified using LI-COR or ImageQuant. All input protein was normalized to actin in the same gel as a control for loading. All biotinylated lanes were normalized to PrPc and/or EAAT1—identical results were obtained with either loading control.

### Antibodies

We used the following antibodies at the titer indicated: VGLUT1 (1:2000), VGLUT2 (1:1000), VGAT (1:2000), SV2 (Developmental Studies Hybridoma Bank, 1:1000), synaptophysin (Sigma, 1:2000), synaptotagmin 1 (Synaptic Systems, 1:1000), VAMP2 (Synaptic Systems, 1:1000), syntaxin 1 (Sigma, 1:1000), SNAP25 (Synaptic Systems, 1:1000), munc18 (BD Biosciences, 1:500), GluR1 (Millipore 1:1000), GluR2 (Millipore, 1:500), NR1 (BD Biosciences, 1:1000), PSD-95 (Neuromab, 1:1000), TfR (Invitrogen, 1:500), rab3 (Synaptic Systems, 1:500), rab5 (Synaptic Systems, 1:500), rab7 (Sigma, 1:500), LAMP1 (Developmental Studies Hybridoma Bank, 1:500), V-ATPase H subunit (Santa Cruz, 1:100), Na^+^/K^+^ pump (Abcam, 1:1000), α-ATP synthase (Abcam, 1:1000), GFAP (Zymed, 1:250), CPE-c term (P. Loh, at 1:500), actin (Millipore, 1:1000), FLAG (Sigma, 1:2000), voltage-gated calcium channel subunit α_1_A (Synaptic Systems, 1:500), voltage-gated calcium channel subunit α_2_δ-1 (Sigma, 1:1000), EAAT1 (Synaptic Systems, 1:1000) and PrPc (S. Prusiner D13, 1:1000). Infrared dye-conjugated secondary antibodies (LI-COR) were used for western blotting at 1:50,000 with the exception of the calcium channel subunit α_1_A and PrPc, which required chemiluminescence detection using secondary antibody conjugated to horseradish peroxidase at 1:5000 (GE Healthcare).

### Immunoblotting

Western blots were quantified using the Odyssey system (LI-COR), with the exception of calcium channel subunit α_1_A and PrPc HRP signal which were acquired by direct detection using ImageQuant. Briefly, tiff files of imaged gels including the proteins of interest and actin were analyzed in ImageJ, subtracting the background for each channel, measuring average pixel intensity for each band (in a fixed area), and normalizing each sample to actin. Results are presented as mean ± s.e.m. for 4–6 animals/genotype. The significance of the difference was assessed by Student’s *t* test.

### Synaptic vesicle transport assays

Synaptic vesicles were purified as previously described^14^. The standard buffer for glutamate uptake contained 148 mM choline or potassium gluconate, 2 mM choline or KCl, 4 mM MgATP, 10 mM HEPES-Tris, pH 7.4, 1 mM choline glutamate, and 40 μCi/ml [^3^H]l-glutamate (Perkin-Elmer). The standard buffer for γ-aminobutyric acid (GABA) uptake contained 145 mM choline or potassium gluconate, 5 mM KCl, 4 mM MgATP, 10 mM HEPES-Tris, pH 7.4, 50 µM GABA, and 65 μCi/ml [^3^H]GABA (Perkin-Elmer). Transport was initiated by adding 100 μg LP2 protein to 200 μl reaction buffer (pre-warmed to 30 °C). The reaction was incubated at 30 °C for 10 min and stopped by rapid filtration and washing four times with 2 ml cold reaction buffer lacking glutamate/GABA. Bound radioactivity was detected by liquid scintillation, and background transport measured in the presence of 100 μM Evans Blue (glutamate) or 5 μM nigericin + 20 μM valinomycin (GABA) was subtracted. In every experiment, each condition was assayed in triplicate and at least three independent experiments were performed using at least three different synaptic vesicle preparations. Results are presented as mean ± s.e.m and the statistics analyzed by two-way ANOVA test with Bonferroni post hoc comparison.

### Electrophysiology

Acute hippocampal slices (350 μm thickness) were prepared from 3- to 4-week-old KO and WT mice. Animals were deeply anesthetized by exposure to isoflurane and decapitated immediately. The brain was rapidly removed and immersed in ice-cold, aerated (95% O_2_–5% CO_2_) slicing solution (containing in mM, 228 sucrose, 2.5 KCl, 1 NaH_2_PO_4_, 7 MgSO_4_, 0.5 CaCl_2_, 26 NaHCO_3_, and 11 dextrose). Vibratome (Leica VT1000S)-cut slices were transferred to artificial cerebrospinal fluid (aCSF) (containing in mM, 119 NaCl, 2.5 KCl, 1.3 MgSO_4_, 2.5 CaCl_2_, 26 NaHCO_3_, 1 NaH_2_PO_4_, and 11 dextrose; 315 Osm; pH 7.4) at 35 °C and allowed to recover for a minimum of 1 h before recording. Hippocampal slices were visualized using an upright infrared differential interference contrast microscope (Olympus BX50WI) and perfused with standard aCSF at room temperature while recording. Electrical stimulation (100 μs) pulses were generated by Master-8 and isolator (W.P.I.), and delivered through a bipolar metal electrode (FHC MX21AEW). For field potentials, synaptic strength was quantified as the initial slope of the evoked response using aCSF-filled microelectrodes (1–2 MΩ). For whole cell recording of evoked EPSCs and miniature EPSPs, the pipette solution contained (in mM) 136 CsMeSO_4_, 7 CsCl, 0.25 EGTA, 10 HEPES, 2 MgATP, 0.3 Na_2_GTP, 8 NaCl, 0.1 spermine, and 7 phosphocreatine. QX314 (5 mM) was added to the internal solution to block sodium channels. For miniature IPSC recording, CsCl in the pipette solution was increased to 75 mM to magnify the GABA response at −70 mV. Tetrodotoxin, picrotoxin (PTX), or CNQX and APV were added to the standard external solution to distinguish miniature EPSCs and IPSCs, respectively. For the measurement of evoked EPSC responses, 20 to 60 sweeps were averaged. AMPAR-EPSCs recorded at a holding potential of −70 mV were measured as the peak amplitude; NMDAR currents in compound EPSCs recorded at 40 mV were measured as the current 100 ms after stimulation. Results are presented as mean ± s.e.m. The results were analyzed by either Student’s *t* test or two-way ANOVA.

Organotypic hippocampal slice cultures were made as previously described^80^. Slices from P6-P8 NHE9^fl/fl^ mice were biolistically transfected with a Cre-mCherry fusion^81^ and cytoplasmic GFP at DIV 2. Recordings were performed at DIV 19–20. Dual whole cell recordings in area CA1 were performed by simultaneous recording from a fluorescent-transfected neuron and neighboring-untransfected control neuron. Pyramidal neurons were identified by morphology and location. Series resistance was monitored online, and recordings in which series resistance increased to >30 MΩ or varied by >50% between neurons were discarded. Dual whole cell recording of evoked EPSCs used an external solution bubbled with 95% O_2_/5% CO_2_ consisting of (in mM) 119 NaCl, 2.5 KCl, 4 CaCl_2_, 4 MgSO_4_, 1 NaH_2_PO_4_, 26.2 NaHCO_3_, and 11 glucose. PTX of 100 μM was added to block inhibitory currents and 1–2 μM 2-chloroadenosine was used to control epileptiform activity. Intracellular solution contained (in mM) 135 CsMeSO_4_, 8 NaCl, 10 HEPES, 0.3 EGTA, 5 QX314-Cl, 4 MgATP, 0.3 Na_3_GTP, and 0.1 spermine. A bipolar stimulation electrode (FHC) was placed in stratum radiatum, and responses were evoked at 0.2 Hz. Peak AMPAR currents were recorded at −70 mV, and NMDAR current amplitudes 100 ms following the stimulus were recorded at +40 mV. Paired-pulse ratio was determined by delivering two stimuli 40 ms apart and dividing the peak response to stimulus 2 by the peak response to stimulus 1.

### Golgi stain

P21 mice were euthanized by CO_2_, their brains dissected, and immediately immersed in rapid Golgi stain reagents (FD Neurotech) following the manufacturer’s instructions. Briefly, the brains were incubated in solution A/B for 3 weeks, cryoprotected in solution C for 48 h, flash frozen in isopentane, and stored at −80 °C. Sections (100 µm) were cut with a cryostat (Leica CM3050S), mounted onto slides coated with 3% gelatin, air dried for 24 h, stained, dehydrated following the manufacturer’s instructions, and mounted using Permount. Single plane images of spine segments in CA1 were collected 60–100 µm from the soma using a Leica DMRB microscope with Leica PL FLUOTAR ×100/1.30 DIL oil objective. The images were used only if >25 spines remained in focus per segment. Image acquisition and analysis were performed by different experimenters, with analysis blind to genotype. The results are presented as mean ± s.e.m. The data were analyzed by two-way ANOVA with Bonferroni post hoc comparison.

### Data availability

The data that support the findings of this study are available from the corresponding author upon reasonable request.

## Electronic supplementary material

Supplementary Information
