# Supplementary material for: A mouse model of autism implicates endosome pH in the regulation of presynaptic calcium entry
Source: Nat Commun. 2018 Jan 23;9:330. doi: 10.1038/s41467-017-02716-5 (PMC5780507; doi:10.1038/s41467-017-02716-5)
Supplement: Supplementary file 1 — Supplementary Information [file 41467_2017_2716_MOESM1_ESM.pdf]

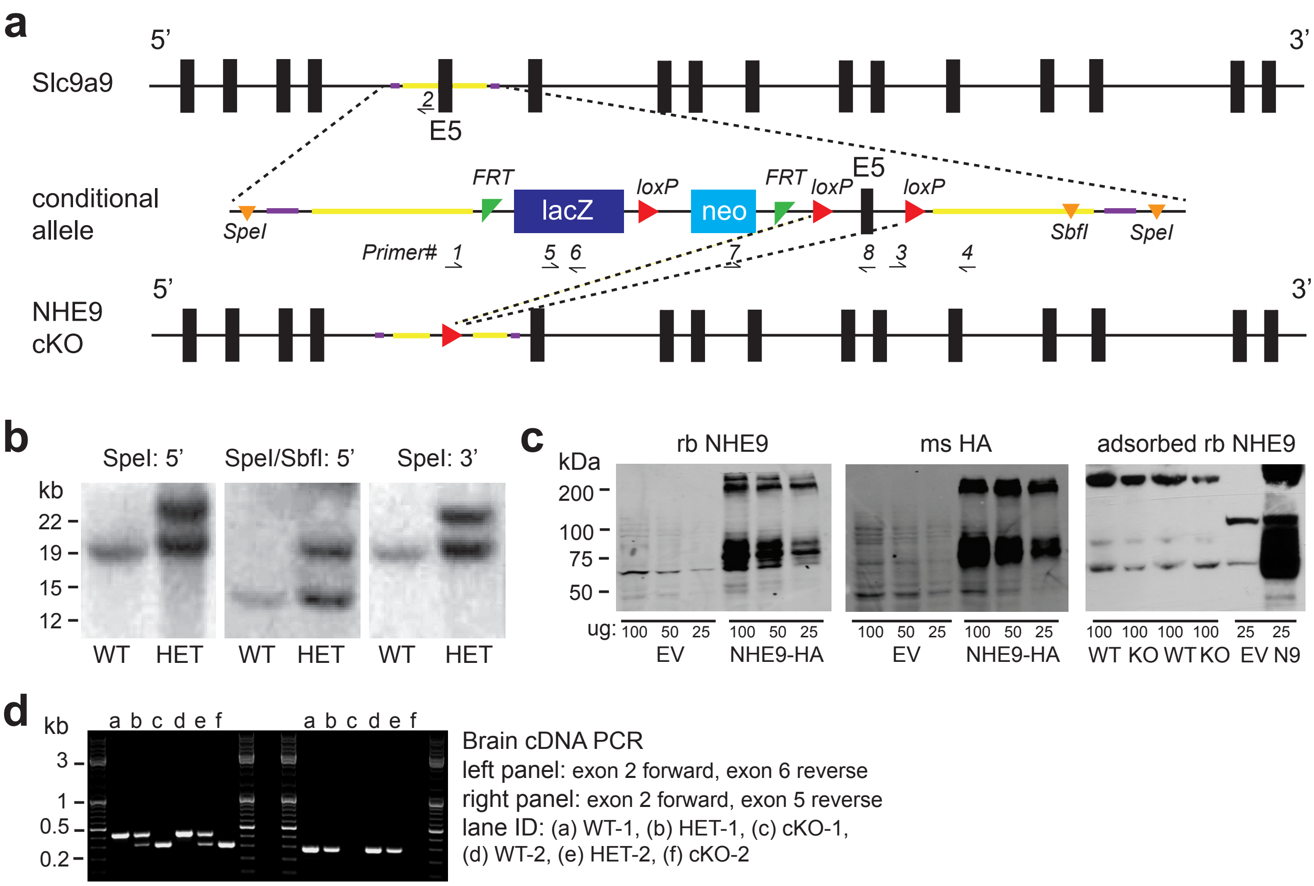

Supplementary Figure 1. Conditional Inactivation of NHE9

(a) Organization of the Slc9a9 gene locus in Mus musculus, with exon 5 (E5) indicated and the homology arms used for recombination shown in yellow (top). The middle panel shows the predicted conditional allele after homologous recombination, with SpeI and SbfI restriction sites (orange arrowheads), lacZ and neomycin resistance genes indicated. The probes used for Southern analysis are indicated in purple, the FRT sites in green and the loxP sites in red. The numbers indicate the position of 8 primers used for genotyping. The bottom panel shows the final state of the allele after recombination by both FlpE and cre recombinase.

(b) Southern blot of DNA from WT and heterozygous mice using probes outside the 5' and 3' homology arms confirms homologous recombination of one allele.

(c) Affinity purified antibodies specific to mouse NHE9 do not detect endogenous NHE9 protein. Left, rabbit anti-NHE9 antibody (a generous gift from J. Orlowski, McGill) specifically detects mouse NHE9-HA in extracts (25-100 µg protein) from cells transfected with NHE9-HA but not empty vector (EV). Middle panel shows an immunoblot of the same samples probed with HA antibody, confirming the identity of the protein as NHE9. Since western blotting revealed no specific bands in WT relative to KO brains (data not shown), we adsorbed the NHE9 antibody with an acetone extract made from the brains of NHE9 KO mice. Right panel, Membrane-enriched extracts (25 or 100 µg protein) from the brains of the same WT and KO mice used for qPCR transcript analysis in Figure 1 were immunoblotted with affinity-purified NHE9 antibody and detected with femto ECL substrate. Adsorbed antibody detects NHE9-HA from HEK cells but shows no specific signal in the brain lysates. We similarly adsorbed several commercial NHE9 antibodies as well as those generated in other labs, all of which could detect NHE9-HA over-expressed in HEK cells, but did not find any capable of detecting a protein in the WT that was not also present in the KO (data not shown). Immunofluorescence similarly failed to identify a signal in the WT that was not also present in the KO (data not shown).

(d) qPCR amplification of exon 2-5 from brain cDNA shows a single product for WT mice, two for HET and one for cKO (left panel). Deletion of exon 5 results in bands 115 bp smaller. PCR from exon2-5 illustrates the absence of a detectable transcript present in cKO brains (right panel).

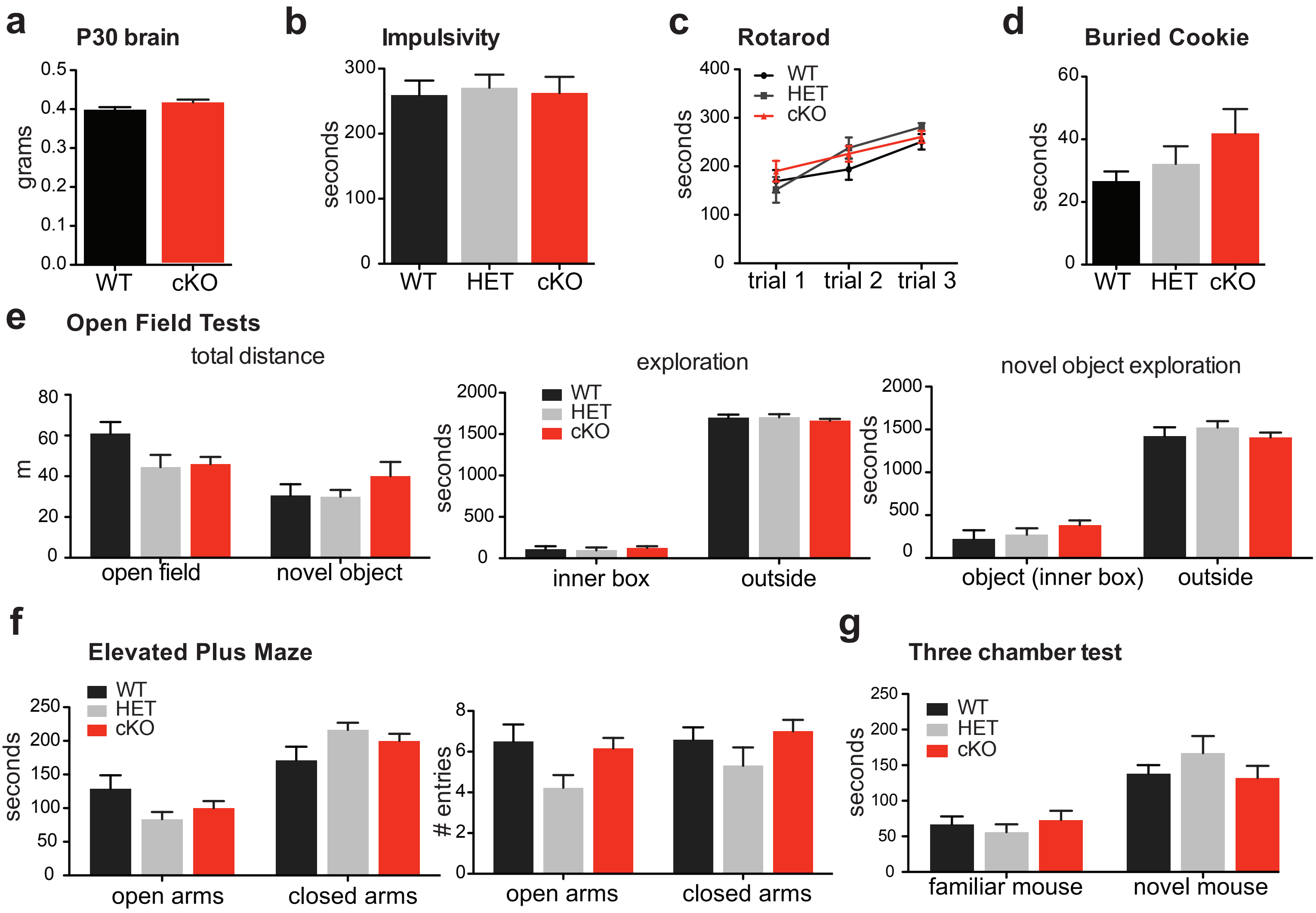

h

		WT (flox/flox)	cKO (-/flox, +nestin-cre)	KO (-/-)
**Body Weight (g)**				
	4-6 month	27.2 +/- 0.5, n=12	24.0 +/- 0.4, n=11, p<0.001***	28.0 +/- 0.6, n=5, NS
**Juvenile Play (s)**				
	soliciting	30.7 +/- 3.0, n=12	9.3 +/- 2.9, n=11, p<0.001***	10.4 +/- 1.6, n=5, p<0.001***
	affiliative	569 +/- 88, n=12	249 +/- 83, n=11, p<0.05*	314 +/-110, n=5 NS
	investigative	79.08 +/- 6.0, n=12	21.64 +/- 7.5, n=1, p<0.001***	26.8 +/- 6.4, n=5, p<0.001***
**Social Recognition (s)**				
	M1 t60’	17.4 +/-3.3, n=10	43.2 +/-5.8, n=10, p<0.01**	39.8 +/-4.7, n=8, p<0.05*
	M2 t120’	41.5 +/-9.3, n=10	30.0 +/-4.5, n=10, NS	41.3 +/-2.4, n=8 NS
**Marble Burying (#)**				
		6.5 +/-1.8, n=13	15.6 +/-0.9, n=31, p<0.0001****	14 +/-2.0, n=6, p<0.05*
**Odorant Investigate (s)**				
	water	3.2 +/-0.8, n=17	5.6 +/-1.8, n=28 NS	7.1 +/-1.8, n=9 NS
	almond	3.4 +/-0.6, n=17	11.3 +/-1.1, n=28, p,0.05*	9.1 +/-1.2, n=9 NS
	banana	1.3 +/-0.7, n=17	10.5 +/-1.7, n=28, p<0.01**	11.1 +/-3.4, n=9, p<0.05*
	male	17.7 +/-3.3, n=17	16.7 +/-2.1, n=28, NS	22.7 +/-3.9, n=9 NS
	female	18.4 +/-2.8, n=17	11.6 +/-2.0, n=28, NS	18.0 +/-2.8, n=9 NS
	bobcat urine	4.7 +/-1.0, n=9	16.2 +/-2.3, n=10, p<0.001***	12.9 +/-2.1, n=9, p<0.05*
**Heat Threshold (s)**				
		25.8 +/-1.8, n=7	20.0 +/-1.2, n=27, NS	17.5 +/-1.5, n=14, p<0.01**
**Adult Grooming (s)**				
		207.5 +/-33.4, n=12	78.6 +/-27.81, n=10, p<0.05*	188.6 +/-45.04, n=10, NS

Supplementary Figure 2. NHE9 cKO Mice Exhibit Normal Locomotor Activity, Anxiety and Olfaction.

(a) cKO brain weight (n=16) is the same as WT (n=22). $p>0.05$ by Student's t-test.
(b) All genotypes showed similar, low impulsivity in the cliff test. WT, n=17; HET, n=18; cKO, n=15
(c) Performance on the rotarod over multiple trials showed no difference in baseline coordination or motor learning between the genotypes. WT, n=14; HET, n=12; cKO, n=20
(d) The buried cookie test shows that basic olfaction remains intact in NHE9 HET and cKO mice. WT, n=14; HET, n=16; cKO n=21
(e) Open field activity levels and exploration do not differ in WT, HET and cKO mice. Left, total distance explored in both the open field and open field with a novel object was determined using Ethiovision tracking software. For the same two experiments (middle and right panels), the time spent in the inner box versus outer perimeter of the arena was also measured and found not to differ. WT, n=10; HET, n=13; cKO, n=21
(f) Behavior in the elevated plus maze shows no difference in the NHE9 cKO. All genotypes explored the open and closed arms for similar amounts of time (left) with similar entry numbers (right). WT, n=10; HET, n=13; cKO, n=21
(g) In the three chamber test, NHE9 cKO mice show greater preference for a novel over a familiar mouse. WT, n=10; HET, n=10; cKO, n=12
(h) Summary comparison of WT, cKO and full NHE9 KO (-/-). cKO and KO differ only in body weight. Comparison of cKO to WT or KO to WT was performed by one-way ANOVA with Dunnets post-hoc test, with N variable to each test. Data indicate mean ± s.e.m.

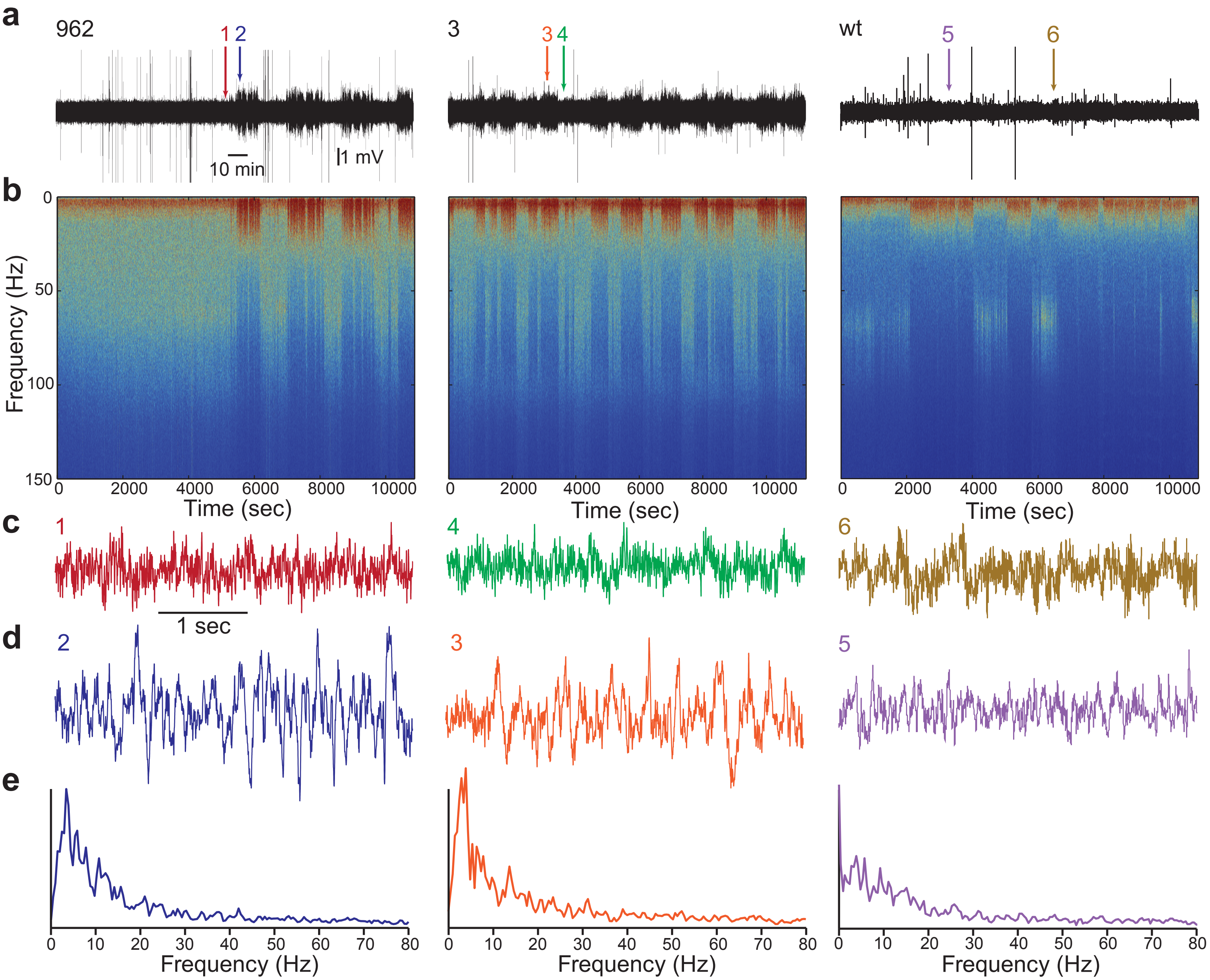

Supplementary Figure 3. NHE9 Knockout Mice Exhibit Prolonged High Voltage Spikes
Interhemispheric EEG recordings were made over the prefrontal cortex of two freely moving NHE9 knockout (962 left and 3 middle) and one wild type C57Bl/6Tn mouse (right). **(a)** Sample EEG records at low temporal resolution show intermittent high voltage activity that lasts up to 10 minutes in the knockouts but not wild type. Arrows and numbers indicate segments of the trace shown at expanded temporal resolution in **(c)** and **(d)**. **(b)** In knockout mice, the corresponding spectrograms show that the bursts of high amplitude activity coincide with periods of increased low frequency (<20 Hz) and decreased high frequency (>30 Hz) power. These episodes also correlate with immobility in the knockouts. Baseline **(c)** and high voltage **(d)** EEG activity are shown at high temporal resolution for the knockout mice (left and middle). Two segments of the trace from wild type are shown for comparison to the right. **(e)** Spectrogram of the high voltage activity in **(d)** shows a peak frequency at 3-4 Hz for both knockout mice, but the wild type shows a peak at 0.

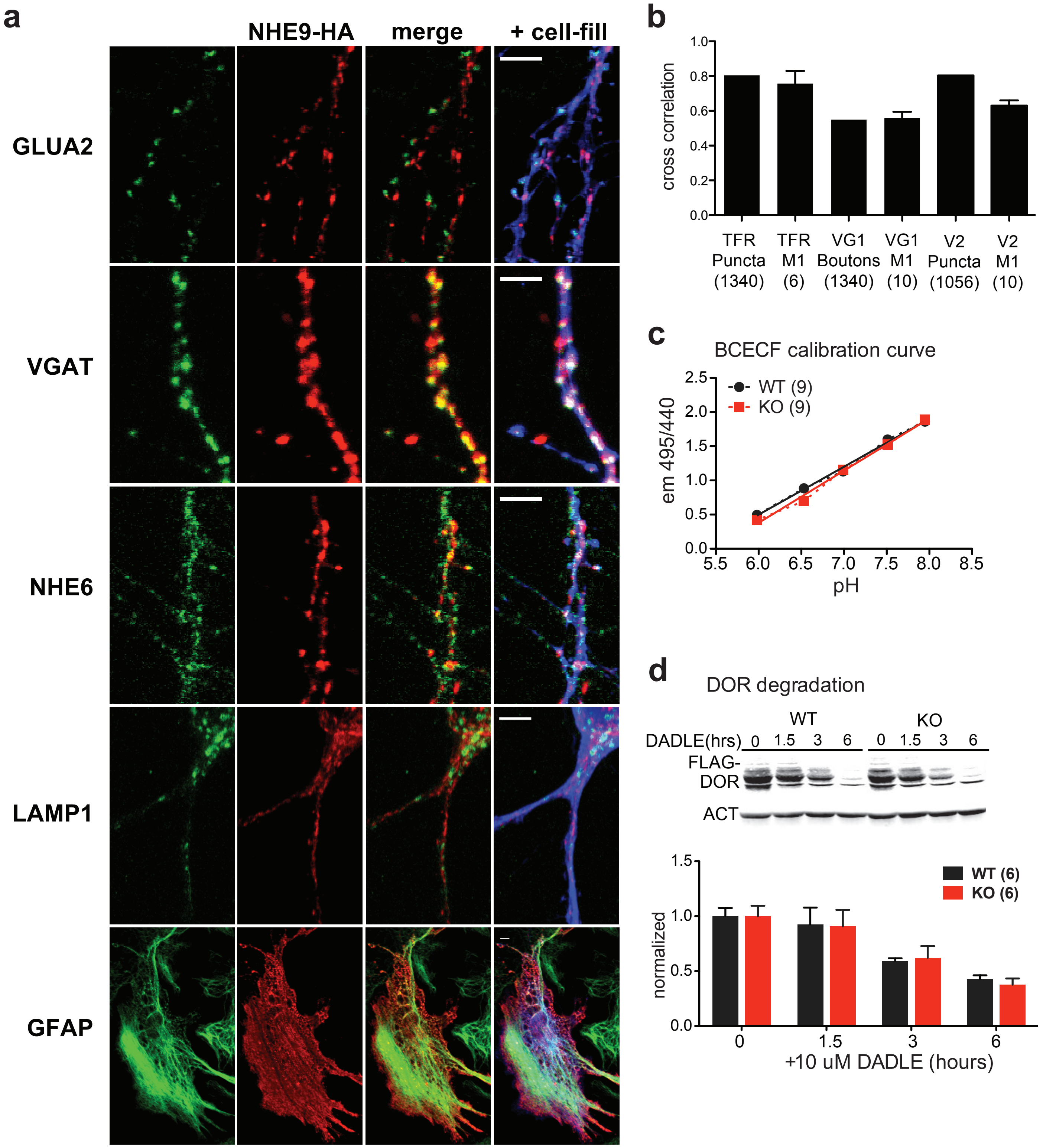

Supplementary Figure 4. Subcellular localization of NHE9, cytosolic pH calibration and endolysomal degradation.

(a) Hippocampal neurons from NHE9 KO mice were cotransfected with EGFP and mouse NHE9 C-terminally tagged with HA (NHE9-HA). DIV14 neurons were fixed and stained for GFP, HA and the endogenous proteins indicated. NHE9 does not colocalize with the postsynaptic protein GluA2 but strongly colocalizes with the vesicular GABA transporter (VGAT). NHE9 colocalizes partially but not completely with the related isoform NHE6. NHE9-HA does not colocalize with the lysosomal protein LAMP1. In astrocytes, NHE9-HA has a diffuse distribution through the cell. Scale bars all indicate 5 µm.

(b) Cross correlation analysis was performed to determine what percentage of TfR+ endosomes, $VGLUT1^{+}$ boutons and $VAMP2^{+}$ punctae that contain NHE9-HA immunoreactivity. n=1056-1340 puncta or boutons, 2-3 cultures Manders cross correlation coefficients (M1) were calculated for the percent TfR^{+}, $VGLUT1^{+}$, or $VAMP2^{+}$ pixels that overlap with NHE9-HA. n=6-10 coverslips, 2-3 cultures

Supplementary Figure 4, continued

(c) BCECF was calibrated using live neurons loaded with BCECF-AM. External solutions at the pH indicated was used to equilibrate vesicle with buffer pH in the presence of ionophores nigericin and valinomycin to equilibrate vesicular with buffer pH. The lines were fit by linear regression, and r2=0.995 for WT, 0.992 for KO. WT, n=9 neurons/3cultures; KO, n=9 neurons/3 cultures

(d) A cDNA encoding the FLAG-tagged, G protein-coupled delta-opioid receptor (DOR) was transfected into hippocampal neurons and the cells at DIV14 stimulated with the agonist DADLE (10 µM) to monitor degradation in the endolysosomal pathway. Western blotting for the FLAG epitope shows that DOR rapidly internalizes in response to DADLE and then undergoes degradation. WT and KO, n=6 wells/3 cultures. Bars indicate mean ± s.e.m.

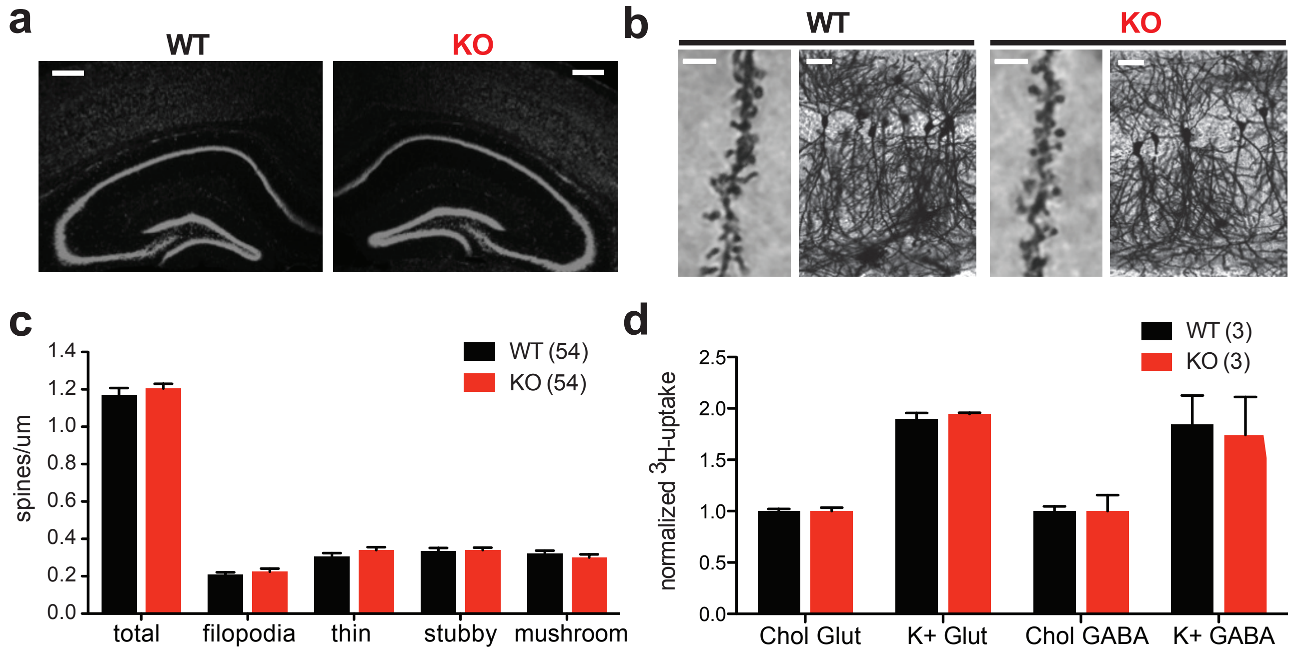

Supplementary Figure 5. Loss of NHE9 Does Not Affect Spine Number, Morphology or Vesicular Glutamate Transport Activity.

(a) Nissl stain of WT and NHE9 KO brain slices shows grossly normal brain architecture. Scale bar 250 µm.
(b) Representative Golgi stain of CA1 dendritic spines and branching shows no difference between NHE9 KO and WT. Scale bars indicate 5 µm for dendritic spines (left) and 25 µm for dendritic branching (right).
(c) The analysis of spine density and shape by electron microscopy shows no difference between WT and KO hippocampal CA1 pyramidal neurons. WT, n=54 neurons/4 brains; KO, n=54 neurons/4 brains
(d) Synaptic vesicles purified from the whole brain of WT and NHE9 KO mice were incubated for 10 minutes with radiolabeled glutamate or GABA in the presence or absence of K^+. The background in Evans Blue was subtracted for glutamate uptake, and in the inophores valinomycin and nigericin for GABA. K^+ stimulation for each LP2 was normalized to the uptake in choline. n=3 independent LP2 preparations.

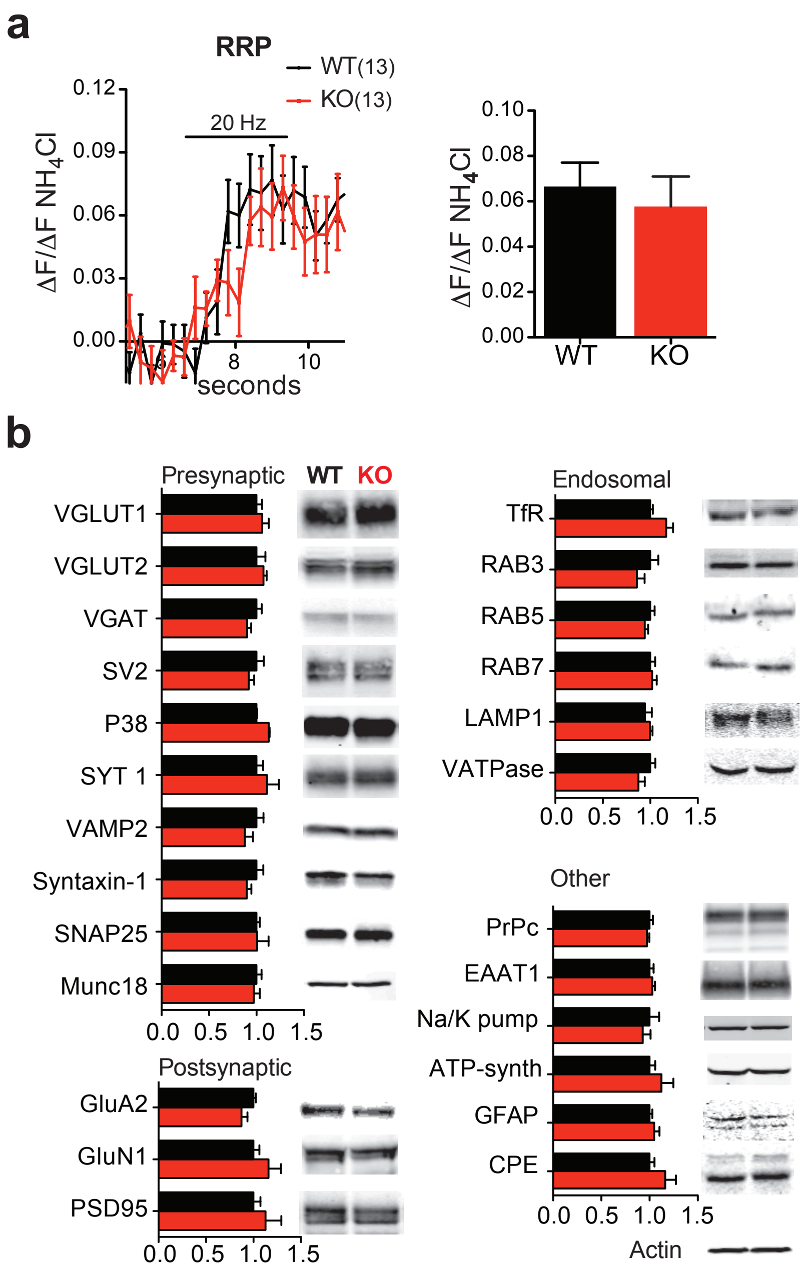

Supplementary Figure 6. Loss of NHE9 Does Not Affect Readily Releasable Pool Size or the Amount of Synaptic Proteins.

(a) Readily releasable pool size was determined as the peak response of VGLUT1-pHluorin (expressed in hippocampal neurons) to 20 Hz stimulation for 2 seconds (right panel). There is no difference between NHE9 KO and WT. WT, n=13 coverslips/3 cultures; KO, n=13 coverslips/3 cultures (b) Extracts from P21 hippocampi were analyzed by fluorescent western blot for presynaptic, postsynaptic, endosomal and other proteins. Loss of NHE9 produced no detectable change in total amount of any of these proteins. Bars indicate mean ± s.e.m. n=4 brains/genotype

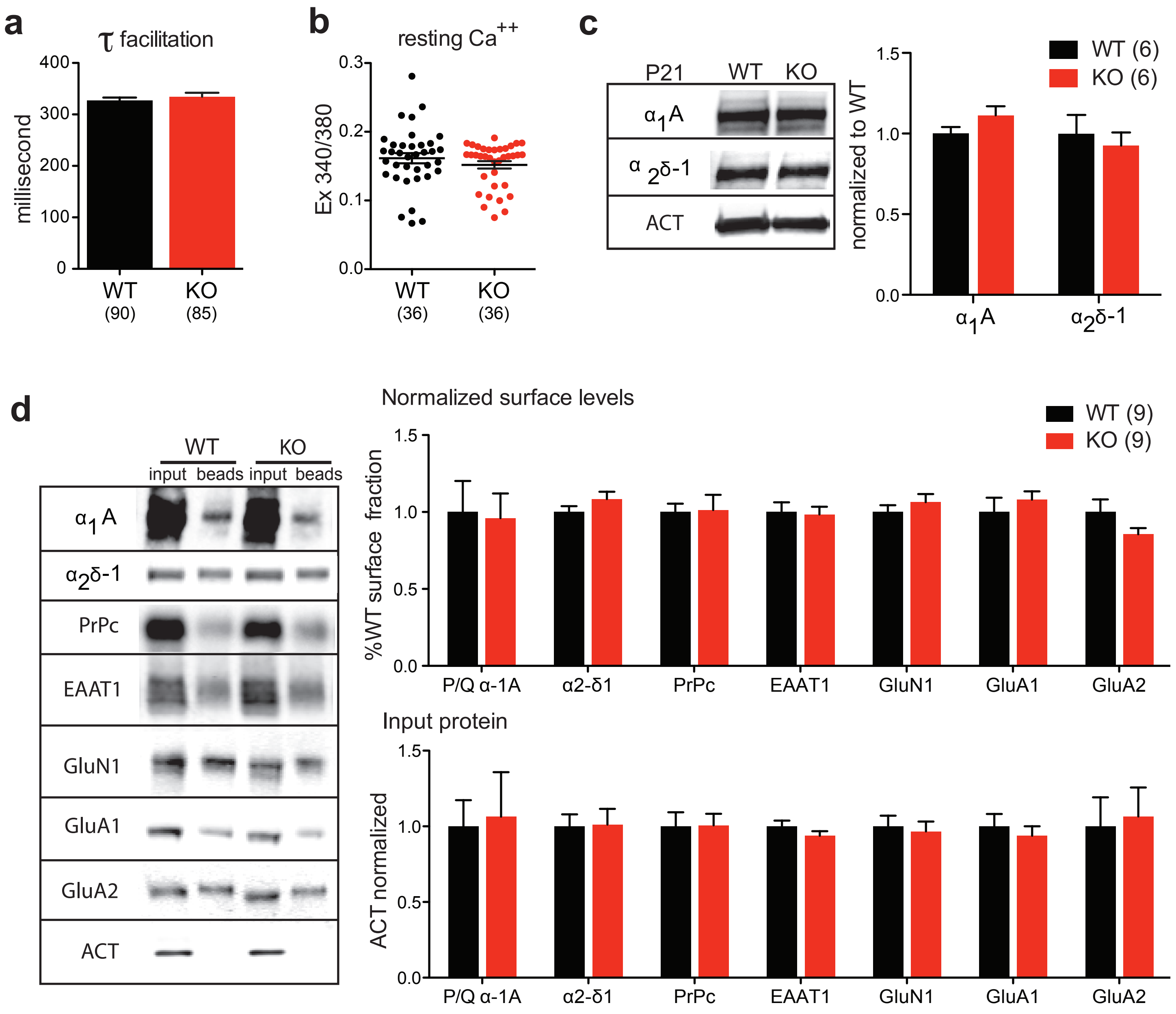

Supplementary Figure 7.

Loss of NHE9 Does Not Affect Facilitation of Ca^{++} Influx, Resting Ca^{++} or the Surface Fraction of Ca^{++} Channel Subunits and Glutamate Receptors.

(a) The time constant was determined by fitting the average paired pulse response (50 ms ISI) to a single exponential decay. n=85-90 averaged paired pulse stimuli from 18-20 coverslips with 30 boutons/coverslip across 3 separate cultures

(b) Baseline Ca^{++} imaging with Fura-2-AM shows no difference between genotypes. p=0.29 by unpaired two-tailed Student's t-test . n= 36 neurons/6 cultures.

(c) Hippocampal extracts from P21 WT and KO mice show no difference in total amount of calcium channel pore-forming subunit α1A or auxiliary subunit α2δ1. Left panel shows a representative western blot, and right panel quantification normalized to actin as loading control, then to WT. No significant difference by Student's t-test. n=6 hippocampi/genotype

(d) Loss of NHE9 does not alter surface levels or total protein in culture neurons. Left panel shows representative lanes from the western blot, right panel the quantification of surface protein fraction expressed as % WT and total protein from the input lysate. No significant difference by Student's t-test. n=9 replicates across 3 cultures/genotype

Data indicate mean ± s.e.m.

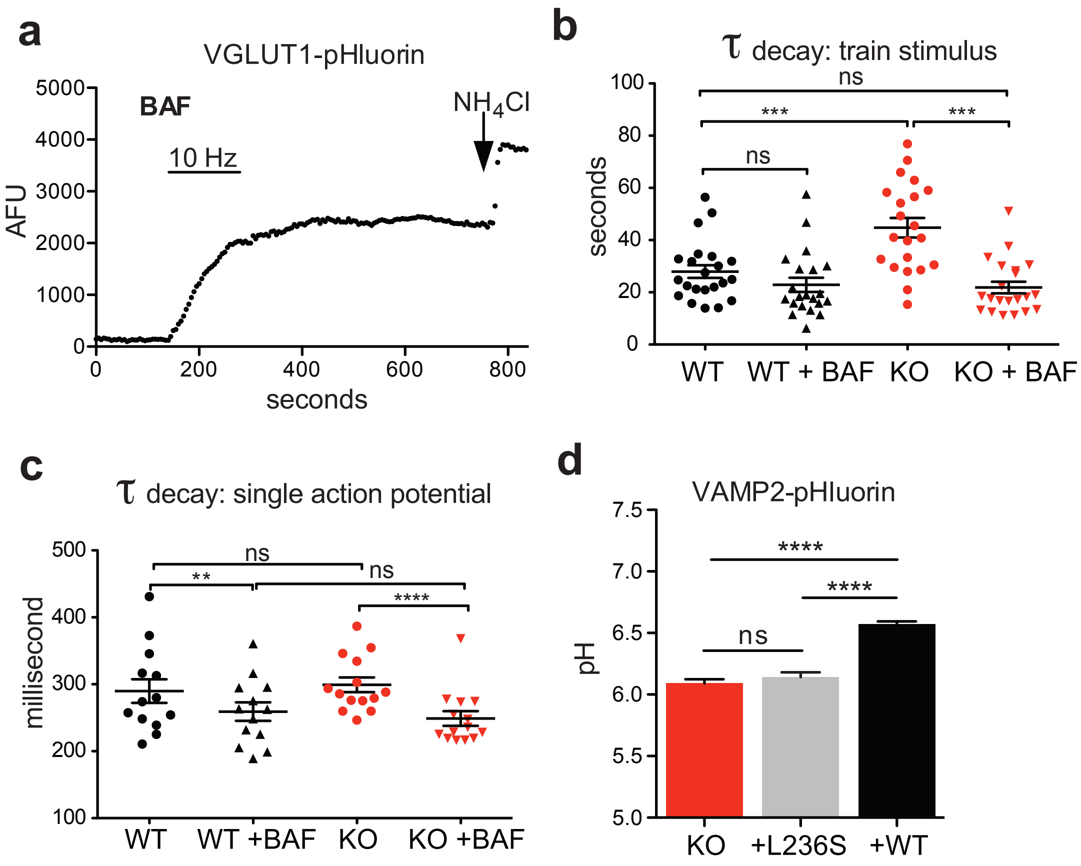

Supplementary Figure 8.

Inhibition of the H^+ Pump accelerates Ca^{++} clearance in boutons.

(a) Representative trace from control experiment to verify that bafilomycin loaded into vesicles expressing VGLUT1-pHluorin increases the pH of these vesicles and the more neutral pH is stable for 10 minutes.

(b) Analysis of the single exponent time constant for Ca^{++} decay after 10 Hz stimulation shows that bafilomycin accelerates decay in the KO neurons. ***, $p<0.001$ by one-way ANOVA with Bonferroni. WT, n=22/21 coverslips/3 cultures; KO, n=21/21 coverslips/3 cultures genotype per condition

(c) Analysis of the single exponent time constant for Ca^{++} decay in response to a single action potential before and after synaptic vesicle loading with bafilomycin shows that bafilomycin accelerates the decay of Ca^{++} fluorescence for both WT and KO neurons (right panel). **, $p<0.01$; ****, $p<0.0001$ by one-way ANOVA with Bonferroni. WT, n=15/15 coverslips/3 cultures; KO, n=14/14 coverslips/3 cultures genotype per condition.

(d) Synaptic vesicle pH in KO neurons transfected for 7 days with empty vector control (KO), NHE9 L236S mutant (+ L236S) or wild type NHE9 (+ WT) plasmids. ****, $p<0.0001$ by one-way ANOVA with Bonferroni. n=99-115 boutons averaged from 2-3 coverslips across 2-3 cultures per transfection. Data in (b) through (d) represent mean ± s.e.m.
